# Ecosystem consequences of multi-trait response to environmental changes in Japanese medaka, *Oryzias latipes*

**DOI:** 10.1093/conphys/coaa011

**Published:** 2020-04-04

**Authors:** Beatriz Diaz Pauli, Eric Edeline, Charlotte Evangelista

**Affiliations:** 1 Department of Biosciences, Centre for Ecological and Evolutionary Syntheses (CEES), University of Oslo, Blindernveien 31, N-0316 Oslo, Norway; 2 ESE Ecology and Ecosystem Health, INRAE, Agocampus Ouest, 65 rue de Saint-Brieuc 35042 Rennes, France

**Keywords:** behaviour, community structure, ecosystem processes, growth, intraspecific biodiversity, stoichiometric traits

## Abstract

Intraspecific trait variation has large effects on the ecosystem and is greatly affected by human activities. To date, most studies focused on single-trait analyses, while considering multiple traits is expected to better predict how an individual interacts with its environment. Here, we used a mesocosm experiment with fish *Oryzias latipes* to test whether individual growth, boldness and functional traits (feeding rate and stoichiometric traits) formed one functional pace-of-life syndrome (POLS). We then tested the effects of among-individual mean and variance of fish functional POLSs within mesocosms on invertebrate community (e.g. zoobenthos and zooplankton abundances) and ecosystem processes (e.g. ecosystem metabolism, algae stock, nutrient concentrations). Stoichiometric traits correlated with somatic growth and behaviours, forming two independent functional POLS (i.e. two major covariance axes). Mean values of the first syndrome were sex- and environment-dependent and were associated with (i) long-term (10 generations; 4 years) selection for small or large body size resulting in contrasting life histories and (ii) short-term (6 weeks) effects of experimental treatments on resource availability (through manipulation of light intensity and interspecific competition). Specifically, females and individuals from populations selected for a small body size presented fast functional POLS with faster growth rate, higher carbon body content and lower boldness. Individuals exposed to low resources (low light and high competition) displayed a slow functional POLS. Higher mesocosm mean and variance values in the second functional POLS (i.e. high feeding rate, high carbon:nitrogen body ratio, low ammonium excretion rate) were associated to decreased prey abundances, but did not affect any of the ecosystem processes. We highlighted the presence of functional multi-trait covariation in medaka, which were affected by sex, long-term selection history and short-term environmental conditions, that ultimately had cascading ecological consequences. We stressed the need for applying this approach to better predict ecosystem response to anthropogenic global changes.

## Introduction

Intraspecific variation refers to any form of trait variation within a species, thus occurring within and among populations (Bolnick *et al*., 2011). Intraspecific variation is still rarely considered in conservation ecology (Mimura *et al*., 2017) despite its crucial role in evolutionary and ecological processes that ultimately shape community structure and ecosystem functioning (Bolnick *et al*., 2011; Hendry, 2017). It is vital to understand how anthropogenic activities modulate intraspecific variation (Allendorf and Hard, 2009; Fugère and Hendry, 2018; Pelletier and Coltman, 2018) but also what the ecological consequences of this human-induced change are for developing effective conservation and management plans (Ward *et al*., 2016; Mimura *et al*., 2017; Pelletier and Coltman, 2018). Indeed, variation in phenotypic traits within consumer or producer species can have cascading ecological effects on lower or higher trophic levels in magnitudes similar or stronger than changes in presence/absence of the species itself (Raffard *et al*., 2017; Des Roches *et al*., 2018; Evangelista *et al*., 2019). However, most studies up to date focused on effects of mean values of a single trait when evaluating potential ecosystem consequences (Palkovacs *et al*., 2012), neglecting two key features: trait variance within a population and the interacting effects among multiple traits (Violle *et al*., 2012; Mimura *et al*., 2017). Assessing both multiple traits and trait variance in addition to trait means will improve our prediction on the fate of populations and communities under human environmental change, as they can determine different ecological effects (Violle *et al*., 2012; Ward *et al*., 2016; Mimura *et al*., 2017).

Links between life-history, behavioural and physiological traits are common (Peiman and Robinson, 2017)—sometimes referred to as pace-of-life syndromes (POLSs; Réale *et al*., 2010). Such multi-trait covariation is often assumed adaptive and susceptible to respond to selective pressures (Peiman and Robinson, 2017; Wright *et al*., 2019), but little is known about its causes and consequences (Peiman and Robinson, 2017). Traits can covary within individuals in a population due to genetic correlations or correlational selection (Reznick *et al*., 2000; Peiman and Robinson, 2017). Comparative analysis of within-population trait covariation under different environmental conditions may help disentangling between these two mechanisms (Peiman and Robinson, 2017). Traits can be genetically correlated (i.e. determined by the same genes), when trait covariation is maintained under the different environments, or correlated through correlational selection (i.e. determined by specific environmental selection), when trait covariation only appears under the specific environmental conditions that created the selective pressure (Peiman and Robinson, 2017).

Multi-trait covariations make it complex to predict the ecological impacts of human-induced changes on intraspecific variation (Hendry *et al*., 2011; Mimura *et al*., 2017; Pelletier and Coltman, 2018). For instance, fishing is commonly size selective against large-bodied individuals, leading to an average decrease in body size and faster life histories (Heino *et al*., 2015), but at the same time it can select on other traits such as behaviour (Diaz Pauli and Sih, 2017). We can describe, for instance, at least three different scenarios where life history and behaviour can covary under fishing selection. First, active gears preferentially target large-sized and shy individuals and may lead to fast life history and boldness (Andersen *et al*., 2017; Diaz Pauli and Sih, 2017). Second, passive gears would instead lead to fast life history (by targeting large fish) and shyness (by targeting bold individuals), but the selection for fast life history would be less pronounced here relative to that with active gears because shyness is associated with slow life history (Andersen *et al*., 2017). In the first case, selections on life history and behaviour reinforced each other, while in the second case they mitigated each other’s actions, resulting in different trait covariations due to correlational selection. Third, size selection alone can affect for example larval viability and foraging efficiency due to genetic correlation between these traits and size, as observed in Atlantic silversides (Munch *et al*., 2005; Walsh *et al*., 2006). Thus, the overall fishing impact on a population considered for conservation plans and its effects on recruitment and productivity would depend on how many traits are evaluated, the direction of the selection on the traits (whether they reinforce or mitigate each other) and the nature of the among-trait covariation (Law, 2007; Peiman and Robinson, 2017), hampering our ability to predict the actual ecosystem consequences of altered intraspecific variation (Ward *et al*., 2016).

Raffard *et al*. (2017) proposed that evaluating functional effect traits (e.g. feeding rate, nutrient excretion rate and body elemental composition) that directly affect ecosystem processes (Violle *et al*., 2007) and POLS traits (life history, physiology, behaviour) together would improve the assessment of the ecosystem consequences of intraspecific variation. Such functional effect traits may covary with POLS traits through metabolic and stoichiometric constraints (Brown *et al*., 2004). For instance, according to ecological stoichiometry, individuals with a fast pace of life (short life, high activity, fast somatic growth and reproduction) would also display high feeding rate, high body nutrient content (e.g. low C:P and C:N body ratios) and low nutrient excretion rates relative to slow-paced individuals (Sterner and Elser, 2002; Raffard *et al*., 2017). Surprisingly, only few studies evaluated the effect of variation in functional POLS on ecosystem functioning, despite its integrative role in assessing the functional role of individuals (but see Raffard *et al*., 2017).

Using a multiple-trait approach (i.e. growth rate, boldness, feeding rate, ammonium excretion rate and body nutrient content), we evaluated whether (i) all the traits covaried, (ii) a single functional POLS explained most trait variation and (iii) mean and variance values in the POLS within mesocosm modified ecosystem processes (e.g. gross primary productivity, respiration, algae stock and nutrient concentrations) and invertebrate community (i.e. zoobenthos and zooplankton abundances). To do so, we performed an outdoor mesocosm experiment with fish (Japanese medaka, *Oryzias latipes*) originating from two lines that differed in life-history strategy in response to size-dependent selection (Renneville *et al*., 2020). Specifically, selection for small vs. large body size generated two medaka populations with early maturation vs. late maturation, which resembled the life histories presented by fished and non-fished natural populations, respectively (Heino *et al*., 2015). In addition to size-selective fishing mortality, wild fish populations also face human-induced changes in resource availability through changes in bottom-up (i.e. increased nutrients; Hays *et al*., 2005) or top-down forces (e.g. increased predation or fishing; Heino and Godø, 2002). We mimicked changes in resource availability, by exposing medaka to two levels of both light intensity and interspecific competition, which increased (competitor absence and high light) or decreased (competitor presence and low light) resource availability (Taylor *et al*., 2015; Závorka *et al*., 2017). We assessed whether the selected populations with contrasted life histories exposed to different resource availability differed in both among-trait covariation and mean values of the functional POLS. To gain insights on the causal mechanism of such covariation (genetic correlation vs. correlational selection), we compared the patterns of trait covariation within each population among the different environmental conditions (Peiman and Robinson, 2017). Finally, mean and variance values of the functional syndrome were used to evaluate how intraspecific variation in multiple traits affects the ecosystem (Violle *et al*., 2012; Mimura *et al*., 2017).

We expected covariation among behavioural traits, growth rate and stoichiometric traits and that a single functional POLS would explain most of the trait variation. Within each (fast and slow life history) population, we anticipated trait covariation to be maintained under different environments and hence be due to genetic correlation (Walsh *et al*., 2006; Peiman and Robinson, 2017). Individuals with fast life history would present fast functional POLS (Sterner and Elser, 2002; Raffard *et al*., 2017) and exert weaker bottom-up regulation through reduced released of nutrients, but stronger top-down regulation due to their increased rate of prey consumption (El-Sabaawi *et al*., 2016; Leal *et al*., 2017; Raffard *et al*., 2017). Finally, we hypothesized that fish presenting large among-individual variance in POLS would impose more or stronger ecological effects in their mesocosms driven for instance by increase functional diversity and/or increase interspecific interactions, relative to fish with small differences among them (Bolnick *et al*., 2011; Mimura *et al*., 2017).

## Material and methods

### Model species

In the laboratory, two lines of medaka were created differing in size and maturation for 10 generations (see details in Renneville *et al*., 2020). Two lines (hereafter referred to as populations) were selected for either a large or small body size at 75 days post-hatch. We did not use the control line here (i.e. size-independent selection) because our aim was to assess the multi-trait response of populations with opposite life histories. After seven generations of selection, mature individuals from the fast life-history population were 5.5% shorter in standard length at 75 days post-hatch and presented three times higher odds of being mature than those from the slow life-history population (Renneville *et al*., 2020). Ninety-six mature medaka (sex ratio 1:1, four fish per mesocosm; 0.01 fish/L) of similar body size (mean ± SD = 23.2 ± 1.1 mm) from the 11th generation were randomly selected, measured for initial standard length (SL_i_ ± 1 mm), weighed (*W*_i_ ± 0.001 g) and individually marked after anaesthesia (MS-222) with visible implant elastomer (VIE; Northwest Marine Technology, USA) and then transferred to experimental mesocosms in August 2017.

### Mesocosm experimental design

We used a full-factorial design in which we crossed population life history (fast vs. slow), light intensity (high vs. low) and interspecific competition (present vs. absent). Light intensity was manipulated using shade nets with different mesh sizes that allowed the passage of 92% (high light intensity) and 70% of ambient light (low light intensity) and protected each mesocosm from allochthonous inputs. We used nine-spine sticklebacks *Pungitius pungitius* as competitor species (mean ± SD = 40.83 ± 1.2 cm SL) due to their diet preferences for zooplankton and benthic invertebrates (Gross and Anderson, 1984) being similar to medaka’s (Edeline *et al*., 2016). Nine-spine sticklebacks did not predate on adult medaka. Each treatment combination was replicated three times (*n* = 24 mesocosms) and was randomly assigned to a mesocosm following a block design (*n* = 2 blocks).

In April 2017, 24 outdoor mesocosms (500 L, 0.8 m deep, 1.0 m diameter) were installed at the platform PLANAQUA of the CEREEP Ecotron Ile-De-France and were simultaneously filled with 100 L dechlorinated tap water and 300 L of pre-filtered oligotrophic water from a local pond. They were supplied with 2 L of a highly concentrated and homogenized zooplankton mixture (Copepoda and Cladocera), a 2-L sediment mixture (including benthic invertebrates) collected locally, and enriched with 2 mL of a liquid mixture of KH_2_PO_4_ and NaNO_3_ to enhance primary production (Supplementary Information A). Each mesocosm was then given 4 months to mature before fish were introduced.

The experiment started on 7 August when the four fish were introduced into the mesocosms. At the end of experiment (6 weeks later), relevant ecosystem processes and invertebrate abundances were quantified in each mesocosm. Immediately afterwards, medaka were recaptured (*n* = 94) and measured for different traits.

### Medaka trait measurements

Individual absolute growth rate of marked medaka (AGR; mg week^−1^) was calculated using (*W*_f_ − *W*_i_)/*t*, where *W*_f_ and *W*_i_ are the final and initial weight of fish, respectively, and *t* is the duration of the experiment (i.e. 6 weeks).

Boldness was estimated from behavioural trials. Variation in the bold/shy continuum refers to an individual’s reaction to a threat situation (Réale *et al*., 2007), and individuals that sit in the bold extreme of this continuum usually present reduced survival in many animals (Smith and Blumstein, 2008). Here, boldness was the difference between the squared-rooted total time of the behavioural test (i.e. 360 s) and the squared-rooted time spent freezing. Behavioural trials were performed the week before and the week after the mesocosm experiment under controlled laboratory conditions where fish were housed individually in 1-L tanks. Overnight acclimation was allowed before the behavioural assessment. Total time freezing (immobile) after a threat (i.e. netted out of the water for 30 seconds) for 6 min was quantified. The same procedure was replicated the next day so that we used four measurements of boldness per individual (two before and two after the experiment) to estimate behaviour repeatability.

Feeding rate was measured 5 h after boldness trials. Specifically, each fish received three pellets of fish food (Novo GranoMix, JBL) and was observed for 6 min. The total amount of bites per minute was used as a proxy for feeding rate (bites min^−1^). Fish never ate a whole pellet in a single bite. The same procedure was repeated the following day, after fish were starved overnight in order to obtain comparable willingness to feed. As with boldness, we had in total four measurements of feeding rate.

Ammonium excretion rate was quantified immediately after the fish were recaptured from the mesocosms. Specifically, each fish was individually incubated for 40 min in a 1-L plastic bag containing 0.5 L of spring water. Filtered water samples (200 mL) were analysed for ammonium concentration (Supplementary Information A). Individual excretion rates (μg ind.^−1^ h^−1^) were calculated following Vanni *et al*. (2002) and corrected for body mass (Torres and Vanni, 2007). These mass-normalized excretion rates were used for further analyses (hereafter referred to as excretion rate, μg g^−3/4^ h^−1^; Supplementary Information A).

To quantify body nutrient content (C:N and C:P body ratios), all individuals were euthanized with a lethal concentration of MS222, gut removed and frozen in liquid nitrogen. Samples were freeze-dried, grounded into a homogeneous powder and analysed for C, N and P composition. For each fish, body nutrient percentages (%C, %N and %P) of dry mass were quantified (Supplementary Information A) and were then used to calculate molar ratios for C:N and C:P.

### Ecosystem variables

All ecosystem variables were measured at the end of the experiment before the fish were removed. Daily community respiration (CR_24_) and gross primary productivity (GPP) were quantified using diurnal changes in dissolved oxygen concentration (measured with EXO2 YSI multi-parameter probe) following Harmon *et al*. (2009; Supplementary Information A).

Pelagic and benthic algae stocks were estimated *in situ* using chlorophyll-a concentration. Pelagic chlorophyll-a (μg L^−1^) was measured with a multi-parameter probe (EXO2 YSI) placed in the water column. Benthic chlorophyll-a (μg cm^−2^) was measured on three tiles (20 × 20 cm) placed in each mesocosm at the start of the experiment and using a spectrometer (BenthoTorch probe, bbe Moldaenke).

Nutrient concentrations (μg L^−1^) in the water column (i.e. ammonium and soluble reactive phosphorus, referred to as N and SRP, respectively) were quantified after filtering 200 mL of water onto a Whatman GF/C filter (1.2 μm pore size). N was quantified using the same protocol as for fish ammonium excretion rate. SRP was quantified with the ammonium molybdate method.

Sediment nutrient content (C:P and C:N ratios) was obtained by collecting with a hand-net a 10-mL sample of organic matter settled at the bottom of each mesocosm in three different locations equidistant to each other and distant at least by 0.25 cm from the walls of the tank (total 30 mL mesocosm^−1^). Samples were stored at −20°C until being freeze-dried and grounded to a homogeneous powder. C:N and C:P ratios were obtained following the same procedure as with fish body nutrient content.

Zooplankton was sampled by filtering 4 L of water collected from two locations through a 250-μm sieve. Samples were preserved in 70% ethanol and stored-refrigerated, and organisms were counted and identified to the lowest possible taxon (family or order), including Copepoda (i.e. Cyclopidae and Calanidae) and Cladocera (i.e. Bosminidae).

Zoobenthos community was obtained by collecting the sediment in two transects (96 cm × 12.5 cm) with a hand net. These were homogenized and represented 0.24 m^2^ of the bottom of each mesocosm. Samples were then preserved in 100-mL pots and processed as above. They were mainly composed of Chironomidae larvae, Planorbidae, Corbiculidae, Hydrachnidia, Nematoda and Ostracoda.

### Statistical analysis

All statistical analyses were performed using R software (version 3.5.0; R Core Team, 2018).

- Behavioural repeatability

For boldness and feeding rate, adjusted repeatability (*R*_adj_) between measurements (*n* = 4) and confidence intervals (BCI) were estimated with Gaussian univariate mixed effect models, with fish identity as random effect and with the time of testing (before or after mesocosm experiment), sex and body weight included as fixed effects (R package MCMCglmm version 2.29; Hadfield, 2010) following Dingemanse and Dochtermann (2013; Supplementary Information A).

- Among-trait covariation

Covariation among fish traits (i.e. AGR, boldness, feeding rate, excretion rate, C:N and C:P body ratios) was assessed from the complete pooled data set with multi-variate pairwise variance-covariance matrices and associated correlation coefficients. In addition, in order to gain insights on whether traits covary due to genetic correlation or correlational selection, we assessed the patterns of among-trait covariation within each size-selected population (fast and slow life history) at the different environmental treatments and sexes with variance–covariance matrices (*I*) for each single data set. Following Houslay *et al*. (2018), we calculated ‘difference matrices’ (*D*) between pairs of variance–covariance matrices (*I*) by subtracting one matrix from another. We estimated differences between populations (*D*_fast life history vs. slow life history_). In addition, for each population, we compared within-population difference between competition intensity *D*_presence vs. absence competitor_ = *I*_competitor present_ − *I*_competitor absent_), light intensity (*D*_high light vs. low light_) and sex (*D*_male vs. female_), resulting in the evaluation of three differences matrices per population (six in total). Identical *I* matrices will result in *D* matrices with all elements equal to zero and hence no significant difference in among-trait covariation between treatments, indicating trait covariation is non-plastic, which can hint that it is due to genetic correlation (Peiman and Robinson, 2017). Parametric bootstrapping (5000 simulations with R package boot; Canty and Ripley, 2019) was used to estimate 95% confidence intervals around each element of the *D* matrices (Houslay *et al*., 2018). All traits were divided by their global standard deviation prior to analysis, putting each type of trait on a common scale, but conserving any differences across contexts (Houslay *et al*., 2018). In addition, multi-variate mixed effects models (with mesocosm identity as random effect) were performed to disentangle among-individual and among-mesocosm covariation among traits (Supplementary Information B). Results showed that all significant covariations among traits occurred among individuals, while no significant trait covariation occurred among mesocosms. Therefore, we may interpret that any among-trait covariation observed was mainly driven by among-individual covariation and hence represent syndromes (Dingemanse *et al*., 2012).

- Presence of functional POLSs

We performed a principal component analysis to assess the presence of a single POLS, i.e. whether a single major axis of variation could explain most (i.e. > 50%) of the individual variation among fish traits. We used the PCA function in R package FactoMineR (version 1.41; Lê *et al*., 2008) which computes loadings of the traits and also allows to test for Pearson correlation between each trait and the PC axes (Husson *et al*., 2011). Then, we assessed the effect of sex, population life history, light intensity and interspecific competition on each PC axis comparing mean values of the individual scores on each PC axis (i.e. functional POLSs) with *v*-test. Significant differences between a treatment level and the overall mean of the PC scores were indicated by absolute *v*-test values larger than 1.96 (Lê *et al*., 2008). Data were scaled to unit variance. Only behavioural measurements (boldness and feeding rate) taken at the end of the mesocosm experiment (*n* = 2 per behaviour) were averaged and used, to ensure all traits compared were measured at the same time (Dingemanse and Dochtermann, 2013; Supplementary Information B). Boldness and feeding rate were square-root-transformed to normalize their distributions (Husson *et al*., 2011). Importantly, the use of a PCA framework allowed us to extract fish PC scores that were subsequently used to test the effect of functional POLS (i.e. mean and variance) on ecosystem variables.

- Effect of POLSs on ecosystem

In order to evaluate how intraspecific variation in multiple traits affected the mesocosm ecosystem, we extracted most of the trait variation and tested its effect on ecosystem variables. The first two principal components (PC 1, PC 2) of the PCA were chosen for further analyses, as their eigenvalues were larger than 1 (Jackson, 1993). Individual fish scores on PC 1 and PC 2, which represented two independent functional POLSs (see Results section), were extracted. For each mesocosm, we calculated mean and variance of these PC scores (POLSs) and they were included as predictors in mixed effects models (with design block as random factor; R package lme4 version 1.1–17; Bates *et al*., 2015) to test their effects on invertebrate abundances and ecosystem processes (one mixed effects model per ecological variable). Ecosystem processes and invertebrate abundances were modelled with Gaussian distribution and negative binomial error distributions, respectively. Pelagic algal stock was natural log-transformed to fulfil model requirements. Benthic algal stock included mesocosm identity as additional random factor controlling for multiple measurements. *P* values adjusted for false discovery rate were estimated (FDR-adj-*P*) to avoid type I errors due to multiple comparisons (Benjamini and Hochberg, 1995). To avoid multi-collinearity, only mean PC 2 and variance in PC 1 and PC 2 were included as predictors due to significant correlation between mean PC 1 and variance in PC 1 and PC 2 (Fig. S.D.1).

## Results

All traits were highly variable between individuals (Fig. S.C.1). Boldness and feeding rate were moderately repeatable when considering all the trials (boldness: *R*_adj_ = 0.29 [BCI = 0.19–0.41]; feeding rate: *R*_adj_ = 0.20 [BCI = 0.10–0.32]).

**Table 1:** Variance–covariance matrix (*I*) for the complete pooled data set

	AGR	Excretion rate	C:N	C:P	Boldness	Feeding rate
AGR	1.02 (0.69, 1.32)	0.13 (−0.08, 0.34)	0.18 (−0.08, 0.44)	*0.21 (0.01, 0.43)*	**−0.43 (−0.58, −0.28)**	−0.03 (−0.25, 0.2)
Excretion rate	0.13 (−0.08, 0.35)	1.01 (0.66, 1.34)	*−0.13 (−0.3, 0.04)*	0 (−0.22, 0.2)	*−0.17 (−0.37, 0.04)*	−0.05 (−0.29, 0.16)
C:N	0.18 (−0.08, 0.44)	−0.13 (−0.3, 0.07)	1.01 (0.5, 1.49)	**0.22 (0.02, 0.45)**	−0.25 (−0.43, −0.09)	*0.13 (−0.04, 0.3)*
C:P	0.21 (−0.02, 0.44)	0 (−0.2, 0.21)	0.21 (0.02, 0.45)	0.96 (0.59, 1.34)	**−0.18 (−0.37, 0)**	*0.14 (−0.04, 0.32)*
Boldness	−0.44 (−0.65,-0.23)	−0.18 (−0.4, 0.04)	−0.25 (−0.41,-0.09)	−0.18 (−0.35, 0)	1.02 (0.72, 1.28)	*0.21 (−0.02, 0.41)*
Feeding rate	−0.03 (−0.27, 0.2)	−0.05 (−0.27, 0.18)	0.13 (−0.04, 0.31)	0.14 (−0.04, 0.3)	0.21 (−0.03, 0.44)	1.02 (0.79, 1.23)

Diagonal shows trait variances, with between-trait covariances below and the corresponding correlation coefficients above. Brackets contain 95% confidence intervals. Estimates are pairwise covariance and correlation Pearson estimates, and confidence intervals are estimated from parametric bootstrapping (5000 simulations). Bold marks significant 95% confidence intervals (i.e. do not overlap with zero), while those that only overlap with zero by 0.05 are in italics

### Among-trait covariation

The evaluation of among-trait covariation highlighted significant covariation among behaviours, stoichiometric traits and absolute growth rate (AGR). Specifically, boldness was negatively correlated with growth, and C:N and C:P body ratios (Table 1). There was a non-significant trend for a positive correlation between boldness and feeding rate, while non-significant negative correlation tended to occur between boldness and excretion rate (Table 1, Fig. S.C.1).

**Figure 1:**
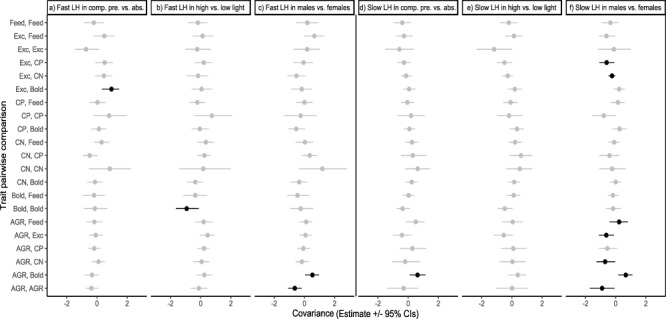
Difference in trait variance–covariance (estimates ±95% CI) comparing the fast life-history population (**a**–**c**) and the slow life-history population (**d**–**f**) within the different contexts: (a, d) presence and absence of a competitor, (c, e) high and low light intensities and (e, f) males and females. Significant differences are in black. AGR = absolute growth rate, Bold = boldness, CN = C:N body ratio, CP = C:P body ratio, Exc = ammonium excretion rate and Feed = feeding rate

Difference matrix evaluation showed no significant differences in the variance–covariance structures within populations due to differences in competition and light treatments (Fig. 1; Table S.C.1). However, there were between-population differences and sex-dependent differences within populations (Fig. S.C.2; Table S.C.2), particularly within the slow life-history population (Table S.C.1f). Fast life-history populations presented higher boldness variance, while slow life-history populations showed higher growth variance and higher covariation of C:N with excretion rate and AGR. Within the two populations, females presented a lower boldness-AGR covariation and a higher growth variation relative to males (Fig. 1c and Table S.C.1). Within the slow life-history population, females also presented an increase in both AGR-C:N and AGR-excretion rate covariations, as well as an increase in covariation of excretion rate with feeding rate, C:P and C:N (Fig. 1d and Table S.C.1). However, differences between populations represented 4 out of 21 pairwise trait comparisons (20%) and the sexual differences represented 2 and 7 out of 21 elements (9 and 33% of changes), for fast and slow life history, respectively.

### Variation within two independent POLSs

Stoichiometric traits were strongly linked to behaviour and life-history traits, thus confirming trait covariation results from the among-trait covariation evaluation. In addition, most trait variation was synthesized into two independent functional POLSs. Specifically, the first syndrome (PC 1) was positively associated with AGR and C:P and C:N body ratios, but negatively associated with boldness (Table 2), as also seen in the trait covariation evaluation. The second syndrome (PC 2) was positively associated with feeding rate and C:N body ratio and negatively associated with excretion rate (Table 2).

**Table 2:** Loadings, correlation coefficient (*r*) of each variable with the PC and *P* values obtained from the principal component analysis (PCA) for the first two principal components: PC 1, PC 2

	PC 1			PC 2		
	Loadings	*r*	*P*	Loadings	*r*	*P*
AGR	0.56	**0.75**	<0.001	−0.10	−0.12	0.273
C:P	0.39	**0.52**	<0.001	0.37	0.42	<0.001
C:N	0.39	**0.52**	<0.001	0.44	**0.51**	<0.001
Excretion rate	0.17	0.22	0.041	−0.47	**−0.54**	<0.001
Boldness	−0.59	**−0.78**	<0.001	0.25	0.29	0.007
Feeding rate	−0.06	−0.07	0.500	0.61	**0.70**	<0.001
Eigenvalues	1.7			1.3		
Cumulative variance	29.5%			51.3%		

Eigenvalues and cumulative variance explained in each of them. Correlation coefficients above 0.5 are highlighted in bold

**Figure 2:**
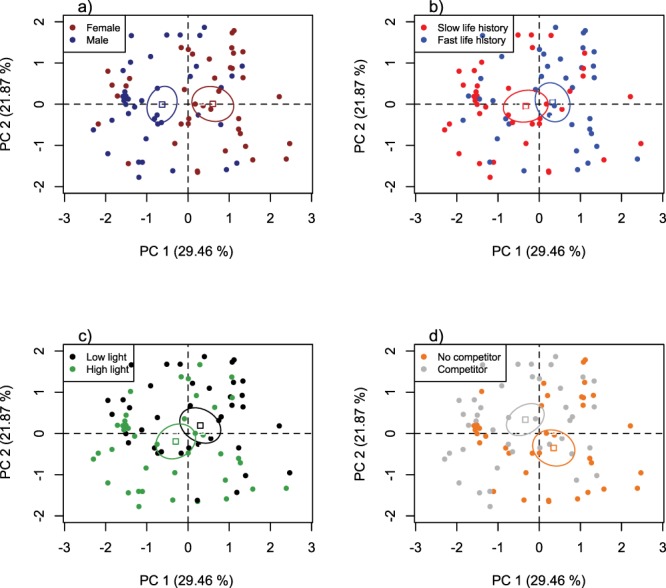
Individual coordinates (dots) and means of each category level (squares) on the first two principal components from the PCA. Ninety-five percent of confidence ellipses around the means are drawn for each category level: (**a**) sex, (**b**) life history, (**c**) light exposure and (**d**) presence of competitor. PC 1 is positively correlated with fish AGR and C:N and C:P body ratios and negatively correlated with boldness. PC 2 is positively correlated with fish C:N body ratio and feeding rate and negatively correlated with ammonium excretion rate

Sex explained 21% of the total variance in PC 1, while population life history and light intensity explained 6 and 5% of it, respectively (Table S.D.1, Fig. 2). Specifically, females and fish from the fast life-history population, as well as fish exposed to low light intensity, on average grew faster, had higher C:P and C:N body ratios and displayed lower level of boldness relative to males (*η*^2^ = 0.21, *v*-test = 4.27, *P* < 0.001; Fig. 2a), fish from the slow life-history population (*η*^2^ = 0.06; *v*-test = 2.26, *P* = 0.023; Fig. 2b) and fish exposed to high light intensity (*η*^2^ = 0.05, *v*-test = −2.06, *P* = 0.038; Fig. 2c), respectively. The presence of a competitor explained 7 and 9% of the total variance in PC 1 and PC 2, respectively (Fig. 2d; Table S.D.1). Within the first syndrome, individuals exposed to a competitor had slower AGR and lower C:P and C:N body ratios, but higher boldness relative to those not exposed to interspecific competition (PC 1: *η*^2^ = 0.07, *v*-test = −2.39, *P* = 0.016). Within the second syndrome, interspecific competition was associated with low excretion rate, but high C:N body ratio and feeding rate (PC 2: *η*^2^ = 0.09, *v*-test = 2.77, *P* = 0.005). The presence of a competitor was thus associated with high or low C:N body ratio depending on the syndrome evaluated. The second POLS was not affected by the other treatments (Table S.D.1).

### Effect of POLS on ecosystem variables

**Figure 3:**
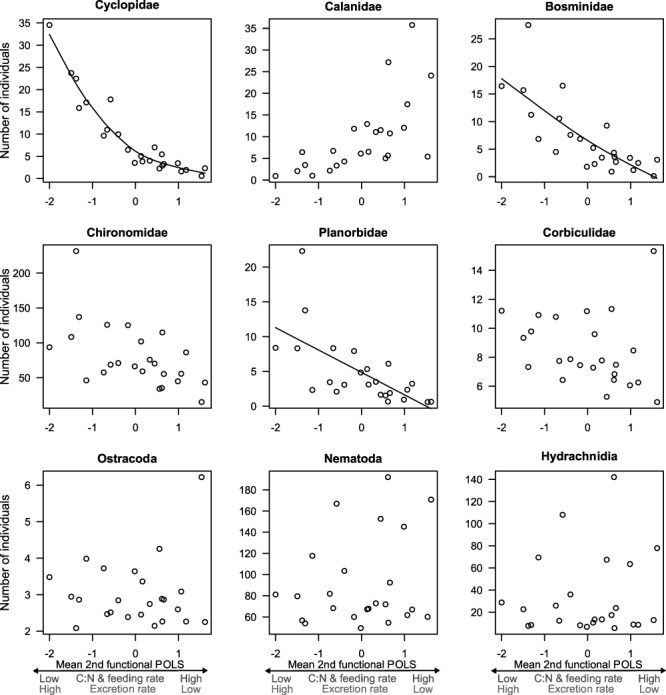
Change in invertebrate abundances relative to mean values in medaka’s second functional POLS (mean PC 2 values within mesocosms). Solid lines represent significant predicted changes

Variation in the second functional POLS affected invertebrate abundances (Table S.E.1), but did not affect ecosystem processes (Table S.E.2). Mean values of PC 2 scores had a significant effect on the abundance of Cyclopidae, Bosminidae and Planorbidae. Specifically, individuals with high feeding rate and C:N, but low excretion rate reduced Cyclopidae, Bosminidae and Planorbidae abundances (Table S.E.1; Fig. 3). In addition, variance (among-individual variance within a mesocosm) in PC 2 scores significantly affected Planorbidae and Hydrachnidia abundances. High variance in feeding rate, C:N and excretion rate decreased abundance of Planorbidae (Table S.E.1) and increased abundance of Hydrachnidia (Table S.E.1, Fig. S3). Variation in PC 1 scores did not affect invertebrate abundances (Table S.E.1) or ecosystem processes (Table S.E.2).

## Discussion

Using a multi-trait approach, our results supported the idea that functional effect traits (i.e. feeding rate, excretion rate and body elemental composition) covary with somatic growth and boldness. This pattern of among-trait covariation (mainly driven by among-individual covariation rather than among-mesocosm covariation) within medaka populations presented slight differences when compared between sexes and size selection, but no differences among short-term environmental conditions. However, we found that medaka presented two independent POLS syndromes. Mean differences in the first POLS (i.e. C:P and C:N body ratios positively correlated with growth and negatively with boldness) were mainly driven by differences between sexes and life-history strategies (fast vs. slow life-history populations) but also by interspecific competition and light intensity. Changes in mean and variance values in the second functional POLS (i.e. C:N body ratio and feeding rate negatively correlated with excretion rate) significantly affected invertebrate abundances, but not ecosystem processes.

### Presence of two functional POLSs

Our results highlighted that multiple-trait associations were linked, forming two functional POLS. As predicted, we consistently found (using among-trait covariation and principal component analyses) that behaviour, growth rate and stoichiometric traits were associated. Specifically, medaka growth rate was energy-driven, as suggested by the positive associations between growth rate and increased C relative to N and P (i.e. high C:N and C:P; Sterner and George, 2000), perhaps indicating that weight gain would be linked to higher investment in C-rich tissues such as fat, which is often involved in fuelling reproduction (Leal *et al*., 2017). In addition, both feeding rate and C:N body ratio were negatively correlated to ammonium excretion rate, as expected by the concept of homeostasis developed in ecological stoichiometry. Indeed, heterotroph individuals with higher nutrient demands are expected to feed more, immobilize more nutrients and excrete less, in order to maintain fixed homeostasis (Sterner and Elser, 2002). The negative association between growth rate and boldness observed here is expected according to the allocation energy management model from the POLS framework, where trade-offs in energy expenditure drive trait covariation (Montiglio *et al*., 2018), as also observed in earlier studies (e.g. Adriaenssens and Johnsson, 2011; Diaz Pauli *et al*., 2019).

The observed differences in the covariation pattern were mainly due to selection, rather than to differences in short-term environmental conditions, which could be interpreted as lack of trait plasticity. The between- and within- population differences (i.e. life histories) in trait covariation were driven by long-term size selection and sex, suggesting that genetic correlation was not the cause of the trait covariation. In addition, these different covariation patterns between life-history strategies (populations) and sexes may indicate that trait covariation is caused by correlational selection and that survival and reproduction are driving such differences (Peiman *et al*., 2017). This contrasts with another experiment on size selection in Atlantic silversides, where the pattern of trait covariation (growth, foraging and larval viability) was due to genetic correlation (Munch *et al*., 2005; Walsh *et al*., 2006). Overall, these comparative analyses remain subjective and cannot definitively conclude why the traits covary and further evaluation is needed (Peiman and Robinson, 2017).

Mean differences in the first functional POLS were mainly associated with sexual differences but also with long-term (size selection induced life history; performed over 10 generations, i.e. 4 years) and short-term (environmental conditions; performed over 6 weeks) manipulations. Specifically, female medaka have higher investment in reproduction and production of eggs (Hasebe *et al*., 2016), which might explain their higher C content relative to males. Probably, this higher investment in reproduction in females relative to males, associated with C-rich tissue and higher weight gain (higher AGR), also entailed a lower investment in costly behaviours such as boldness. Higher boldness in males might be linked to their increased courtship activities relative to females (Sasaki and Yamahira, 2016), which in turn resulted in lower weight gain (AGR) and C content. The long-term size-selection experiment aimed at creating fast life histories also resulted in a faster functional POLS, probably due to selection for earlier maturation, in turn also favouring higher investment in reproduction, high C content, fast weight gain and low boldness (Brown *et al*., 2004; Réale *et al*., 2010). Guppy populations with fast life history presented similar results when considering growth rate, but excreted more ammonium and had similar body ratios compared to slow life guppies (Bassar *et al*., 2010; El-Sabaawi *et al*., 2015; Dalton *et al*., 2017). Thus, trait associations in fish presenting similar life-history strategies seem to be species-specific. Despite both short-term manipulations of resource availability were quite modest (introduction of a single competitor and 20% reduction in ambient light relative to reference treatment levels), we showed that they affected the mean functional POLS. Medaka were bolder and grew slower under interspecific competition and high light conditions, probably due to lower access to resources with the presence of a competitor (Závorka *et al*., 2017) and possibly due to reduced zooplankton growth limited by nutrient-poor algal resources under high-light conditions (Sterner *et al*., 1997; Dickman *et al*., 2008).

Our results highlighted that selection on body size could entail responses of a broad range of traits, maybe due to correlational selection, as the different covariation patterns were driven by size selection and sex and hence probably associated with differential survival and reproduction. However, our results cannot conclusively indicate that size-selective fishing, leading to fast life history (Heino *et al*., 2015), would invariably lead to the same multiple-trait response in commercially exploited fish. Indeed, although a multi-trait response to fishing is expected (Heino *et al*., 2015; Diaz Pauli and Sih, 2017), its direction and magnitude in exploited fish would depend on how the traits are correlated and what is the overall selective pressure imposed. The selective removal of both large-sized and shy fish can exacerbate the response towards early maturation because both should lead to fast life history (Law, 2007; Andersen *et al*., 2017). Alternatively, targeting large-sized and bold fish could attenuate the responses towards fast life history (Olsen *et al*., 2012) or selection on a single trait can affect other genetically correlated traits (Munch *et al*., 2005; Law, 2007). Therefore, more studies on multi-trait response to size-selective fishing are needed to understand its consequences for the population, which will improve the success of management plans (Ward *et al*., 2016; Mimura *et al*., 2017).

### Effect of POL syndromes on ecosystem

Changes in mean and variance values (among individuals within a mesocosm) in the second functional POLS of medaka altered zooplankton and zoobenthos abundances, potentially through direct top-down regulation. Specifically, higher mean values along the second functional POLS (i.e. high feeding rate, high C:N and low N excretion rates) decreased the abundances of zooplankton, likely reflecting direct regulation by predation because these prey are preferentially consumed by medaka (Edeline *et al*., 2016). The reduced abundance of Planorbidae snails is likely explained by ingestion of newly hatched Planorbidae with soft shell, rather than foraging on adult snails because medaka do not possess pharyngeal teeth. However, lower ammonium excretion rate may also reduce biofilm formation (Rubin and Leff, 2007), hence limiting resources for the snails. Thus, medaka may also indirectly reduce snail abundance through bottom-up regulation (e.g. nutrient-mediated). In addition, our results suggested that increasing trait variance among individuals in the mesocosms also resulted in lower snail abundance. The decrease in snail abundance was less steep for increasing values of medaka trait variance than for increasing values of mean trait, probably because the top-down or bottom-up regulation was weaker in mesocosm with more diverse individuals. However, both effects were additive. Moreover, increasing trait variance favoured high abundance of Hydrachnidia. Higher variance in medaka feeding rate may have reduced the top-down regulation on water mites allowing them to reach higher abundance. Alternatively, more diverse mesocosms may impose top-down regulation over a wider range of invertebrate species. Thus, diluting the effect over some species (e.g. Chironomidae) may have allowed more resources for the water mites to thrive (ten Winkel and Davids, 1985). However, whether medaka directly or indirectly affected Hydrachnidia abundances through changes in the relative abundance of other invertebrates cannot be evaluated here as we did not quantify stomach contents. Assessing both the effect of mean and variance values of medaka POLSs revealed more complex interspecific dynamics than relying only on mean trait differences.

Surprisingly, none of functional POLSs modulated ecosystem processes, while previous studies showed that guppy populations with distinct life histories differentially affected ecosystem processes (Bassar *et al*., 2010; El-Sabaawi *et al*., 2015). This could be due to the lower fish density used here (0.01 fish/L) compared to Bassar *et al*. (2010) and El-Sabaawi *et al*. (2015; 0.025 fish/L), although even lower densities of mosquitofish (0.008 fish/L) affected ecosystem processes (Fryxell and Palkovacs, 2017). Another mesocosm experiment (not included in our block design) conducted under similar experimental conditions as the ones in the present study showed that the presence of four medaka per mesocosm resulted in higher levels of whole-system metabolism, algae stock and nitrogen concentrations compared to fishless mesocosms (*n* = 12 controls; Supplementary Information F). Together, our results showed that our density was large enough to affect ecosystem processes, but these were not influenced by variation in the functional POLSs. Instead, ecosystem processes were affected by bottom-up effects (i.e. light intensity; Supplementary Material E). Our objective here was to evaluate the effect of intraspecific multi-trait variation on ecosystem processes by considering both mean and variance values in medaka syndromes. Thus, a lack of effect on ecosystem processes meant that variation within POLSs did not generate ecosystem effects, rather than the two size-selected populations generated similar ecosystem effects.

This study stressed the importance of studying relationships among stoichiometric traits together with life-history and behavioural traits to better predict an individual’s response to environmental changes and its effect on ecosystem functioning (Raffard *et al*., 2017). These results highlighted that rapid changes due to human-induced selection on a single trait (e.g. size or maturation) can entail changes in a whole suite of traits, which in turn had cascading effects into the ecosystem. However, in order to develop successful conservation plans, the causal mechanisms driving among-trait covariation should be better understood.

#### Lay summary

We showed that (i) life-history, behavioural and functional traits in a fish covaried; (ii) they altered the prey community; and (iii) they were affected by environmental stressors. To predict ecosystem consequences of environmental change, we need to assess multiple individual traits because together they define how an individual interacts with its ecosystem.

## Funding

This work was supported by the Research Council of Norway (projects: 251307/F20, 255601/E40), by its mobility program (projects 268218/MO to B.D.P. and 272354 to C.E.) and by the French government (programs: ANR-10-EQPX-13-01 Planaqua, ANR-11-INBS-0001 AnaEE France).

## Supplementary Material

Diaz_Pauli_et_al_Supplementary_material_R2_coaa011
