# Supplementary material for: Ecosystem consequences of multi-trait response to environmental changes in Japanese medaka, Oryzias latipes
Source: Conserv Physiol. 2020 Apr 4;8(1):coaa011. doi: 10.1093/conphys/coaa011 (PMC7125048; doi:10.1093/conphys/coaa011)
Supplement: Diaz_Pauli_et_al_Supplementary_material_R2_coaa011 [file diaz_pauli_et_al_supplementary_material_r2_coaa011.docx]

**Supplementary material**

Ecosystem consequences of multi-trait response to environmental changes in Japanese medaka, *Oryzias latipes*

Beatriz Diaz Pauli^1*^ Eric Edeline^2^, and Charlotte Evangelista^1^

Supplementary information A: Additional material and methods

Supplementary information B: Levels of phenotypic covariation: Multivariate mixed effects models

Supplementary information C: Among-trait covariation

Supplementary information D: Mean and variance values of variation within the functional POLSs

Supplementary information E: Effects of functional POLSs vs. experimental treatment on invertebrate abundances and ecosystem processes

Supplementary information F: Effect of fish absence vs. presence

**Supplementary information A: Additional material and methods**

*Fish rearing conditions in the laboratory*

Fish used in the present study originated from the 11^th^ lab-reared generation and were housed from hatching to adulthood in 3L tanks with 14–17 individuals per tank, keeping the two lines separated. Tanks were maintained in a flow-through system with constant temperature (26ºC) and photoperiod (14:10h L:D). Fish were fed twice daily *ad libitum* with a mixed of living *Artemia salina* and dry food (Gemma Micro 300, Skretting).

*Mesocosms*

In April 2017, 24 outdoor mesocosms (500 L, 0.8 m deep, 1.0 m diameter) were installed at the research station CEREEP – Ecotron Ile de France (Saint-Pierre-les-Nemours, France; www.foljuif.ens.fr). All mesocosms were simultaneously filled on 4–6 April 2017 with 100 L dechlorinated tap water and 300 L of pre-filtered (150 µm mesh size) oligotrophic water from a local pond. They were supplied with 2 L of a highly concentrated and homogenized zooplankton mixture (Copepoda and Cladocera) and a 2-L sediment mixture (including benthic invertebrates) collected locally. Each mesocosm was then given 4 months to mature before fish were introduced. On 12th June, all mesocosms were enriched with 2 mL of a liquid mixture of 0.32 μg P L^-1^ and 6.32 μg N L^-1^ as KH_2_PO_4_ and NaNO_3_, respectively, to enhance primary production.

*Ammonium excretion rate*

Ammonium excretion rate (N-NH_4_^+^) was quantified immediately after the fish were recaptured from the mesocosms. Specifically, each fish was individually incubated for 40 min in a 1-L plastic bag containing 0.5 L of spring water. The spring water was low in N-NH_4_^+^ but had similar characteristic (in particular pH and temperature) than the water from the mesocosms (Paris-Palacios and Biagianti-Risbourg, 2006). Closed bags were placed back in the mesocosm, floating in the water, to maintain temperature constant and to reduce stress during the incubation due to visual contact (Whiles *et al.*, 2009). Filtered water samples (200 ml filtered through Whatman GF/C glass microfiber 1.2 µm pore size-filters) were analysed for ammonium concentration using the fluorometric OPA method in an automated continuous-flow analyser from a single replicate (AA3 autoanalyzer, Seal Analytical, Southampton, UK). Individual excretion rates (ER_i_, μg ind.^-1^ h^−1^) were calculated following Vanni *et al.* (2002): ER_ind_ = (([*N*]_ind_ - [*N*]_C_) × *V*)/*t*, where [*N*]_ind_ and [*N*]_C_ are ammonium concentrations (μg L^-1^) for fish and control bags, respectively, *V* is the volume (L) of spring bottled water in the plastic bag and *t* is the duration of the incubation (h). Control concentrations were obtained from bags (n = 5) filled with spring water but without fish to assess background levels of ammonium. These excretion rates were corrected for fish body weight raised to the power of ¾ as they are likely allometric functions of body weight (Torres and Vanni, 2007). These *mass-normalized* ammonium excretion rates were used for further statistical analyses (hereafter referred to excretion rate, μg g^-3/4^ h^-1^).

1. *Body nutrient content (C:N and C:P ratios)*

To quantify body nutrient content (C:N and C:P body ratios), all individuals were euthanized with a lethal concentration of MS222, gut removed and frozen in liquid nitrogen. Samples were then stored at -20°C until being freeze-dried, grounded into a homogeneous powder and analysed for C, N and P composition. For each fish, %C and %N of dry mass were quantified by dry combustion with a CHN analyser (Flash 200, Thermo Fisher Scientific, Waltham, MA, USA), while %P of dry mass was quantified following the ammonium molybdate method using a spectrophotometer (Uvi Light XT5, Secomam, Alès, France; absorbance measured at 880nm) after digestion with sulfuric acid and potassium persulfate autoclaving at 134ºC for 30 minutes.

1. *Community respiration and gross primary productivity*

Daily community respiration (CR_24_) and gross primary productivity (GPP) were quantified using diurnal changes in dissolved oxygen concentration following (Harmon *et al.*, 2009). Dissolved oxygen concentration (DO) was measured with a multiparameter sonde (EXO2 YSI) three times: at sunrise (*t*_0_), sunset (*t*_1_) and the following sunrise (*t*_2_). Thus, *t*_2_ was then the morning of the end of the experiment, while *t*_0_ was the day before. The difference in DO concentration between sunset (*t*_1_) and sunrise (*t*_2_) multiplied by the water volume in the mesocosm (*V* in L) yielded night community respiration: CR_night_ = (DO*_t_*_1_ – DO*_t_*_2_) × *V*. Daily CR (CR_24,_ mg O_2_) was obtained by multiplying the hourly night respiration rate (CR_night_ divided by 14 hours of night) by 24 (Bott, 2006). GPP (mg O_2_) was obtained by adding CR_night_ to the difference in DO between sunset (*t*_1_) and sunrise (*t*_0_): GPP = CR_night_ + (DO*_t_*_1_ – DO*_t_*_0_) × *V* (Bott, 2006; Harmon *et al.*, 2009).

1. *Statistical analysis of repeatability*

For boldness and feeding rate, adjusted repeatabilities between measurements (n = 4) were estimated with gaussian univariate mixed effects models (R package *MCMCglmm* version 2.29; Hadfield, 2010), with fish identity as random effect and, where the time of testing (before or after mesocosm experiment), sex, and body weight were controlled for following (Dingemanse and Dochtermann, 2013). Repeatability and Bayesian credibility intervals (BCI) were estimated using a Bayesian approach with the default settings of the library MCMCglmm. Convergence of the chains was attained and checked by plotting the MCMCglmm objects generated (Hadfield, 2010).

**Supplementary information B: Levels of phenotypic covariation: Multivariate mixed effects model**

In order to estimate patterns of trait covariation at two distinct levels (among- and within-mesocosm), we performed multivariate mixed effects models (MMMs) with mesocosm identity as random factor (R package *MCMCglmm* version 2.29; Hadfield, 2010). Here, the within-mesocosm variance could be interpreted as among-individuals variance, but we were not able to estimate within-individual variance for ammonium excretion rate, C:N and C:P body ratios and growth rate, as repeated observations of these traits were not available.

It should be noted that we do have repeated observations for boldness and feeding rate which present moderate values of adjusted repeatability (boldness: *R*_adj_ = 0.32 [CI = 0.19 – 0.43], *P*_LRT_ < 0.001; feeding rate: *R*_adj_ = 0.28 [CI = 0.13 – 0.40], *P*_LRT_ = 0.003). Therefore, we decided to use average values of boldness and feeding rate in order to properly estimate covariance among traits at comparable levels following Dingemanse and Dochtermann (2013).

The MMMs were run with the six fish traits as response variable, as well as sex, weight and block identity as fixed effects. Model 1 had mesocosm identity as random effect to estimate among-mesocosm variance. This model also estimated fully unstructured covariance matrices at both the among-mesocosm and within-mesocosm (i.e., among-individual) levels. Following Houslay *et al.*, (2018), three additional models were performed: Model 2 had no random effects, such that whole phenotypic variance (conditional on the fixed effects) was allocated to the residual component (here within-mesocosm variance) and whole among-trait covariance was set to zero; Model 3 contained mesocosm identity as random effect and thus allowed the estimation of among-mesocosm variance but still no among-trait covariance was allowed (neither at among-mesocosm or within-mesocosm levels); finally Model 4 allowed the estimation of among-mesocosm variance and among-trait covariance at the within-mesocosm level. We used Deviance Information Criteria (DIC) comparisons (*Δ*DIC and weights) to provide tests (i.e., across all traits) for (1) among-mesocosm trait variation (Model 2 vs. Model 3), (2) among-trait covariation at the within-mesocosm level (Model 3 vs. Model 4) and (3) significant contribution of among-mesocosm differences to the among-trait covariation (Model 4 vs. Model 1). The model with the lowest DIC value is considered the model that fits the data most appropriately taking into account model complexity (Spiegelhalter *et al.*, 2002). *Δ*DIC_i_ refers to the difference in DIC value between the “best model” and Model_i_, while model weight_i_ estimates refers to the probability of Model_i_ to be the “best model”(Burnham and Anderson, 2002). All models used uninformative priors and with 700 000 iterations, a burn in of 50 000 and thinning of 350 iterations

Model 4 was found to be the best ranked model (Table S.B.1). This allowed for among-mesocosm variation and among-trait covariation at the within-mesocosm level, but constrained among-trait covariation at the among-mesocosm level. The second-best ranked model (Model 1; Table S.B.1) allowed among-trait covariation both among- and within-mesocosm, but none of the pairwise trait correlations at the among-mesocosm level were significantly different from zero (Table S.B.2). Therefore, both models indicated that any among-trait covariation in our data originate at the within-mesocosm level (Table S.B.2 and S.B.3), which can be interpreted as among-individual covariation (see above).

In the main text we evaluated general phenotypic among-trait (co)variation with the associated pairwise correlations in the different populations (fast vs. slow life-history populations). We evaluated differences in the pattern of covariation within populations under different conditions (presence vs. absence light, high vs. low light intensity, male vs. female). In addition, we performed Principal Component Analysis (PCA) instead of MMMs for three reasons:

1) Contrary to PCA, it is not possible to extract scores associated with among-trait covariation from multivariate mixed effects models (referred as BLUPS in MMMs) to test for their effect on ecological variables (Houslay and Wilson, 2017).

2) MMMs fail to detect among-trait correlations when trait repeatability (here among-individuals within mesocosms) is < 0.5 and the number of individuals are < 125 (Dingemanse and Dochtermann, 2013). This becomes an issue particularly when the complete data set is split to compare among- and within-population variation under the different context.

3) Results from the MMMs presented above indicate that general phenotypic among-trait (co)variation is mainly associated with among-individual variation. Although we analysed the general phenotypic covariation to test our different hypotheses due to low sample size, results from MMMs clearly indicate that among-trait covariation is most probably due to among-individual covariation.

| Table S.B.1. Multivariate mixed effects model comparisons based on DIC values, variation in DIC between models and model weights. Descriptions of Model 1 to 4 are given in the text. | | | |
| --- | --- | --- | --- |
| Model | DIC | *Δ*DIC | Weight |
| Model 4 | 1331.5 | 0 | 0.55 |
| Model 1 | 1331.9 | -0.40 | 0.45 |
| Model 3 | 1346.0 | -14.56 | 0 |
| Model 2 | 1399.1 | -67.56 | 0 |

| Table S.B.2. Variance-covariance matrices for a) among- and b) within-mesocosms (i.e., among-individual) obtained from the multivariate mixed effects Model 1. Diagonal shows trait variances, with between-trait covariances below (pale grey) and the corresponding correlation coefficients above. Estimates and 95 % CI (in brackets) are obtained from posterior distributions of the multivariate mixed effects model. Significant 95% CI are highlighted in bold, while marginally significant CI are in italics (i.e., overlap with zero by < **0.05**). | | | | | | |
| --- | --- | --- | --- | --- | --- | --- |
| a) Among-mesocosms matrix | | | | | | |
|  | AGR | Excretion rate | C:N | C:P | Boldness | Feeding rate |
| AGR | 0.4 (0.24,0.85) | 0.07  (-0.4,0.44) | -0.02  (-0.52,0.32) | -0.04  (-0.43,0.42) | -0.24  (-0.55,0.28) | -0.05  (-0.46,0.42) |
| Excretion rate | 0  (-0.29,0.33) | 0.62 (0.34,1.32) | -0.22  (-0.56,0.26) | -0.03  (-0.54,0.33) | 0.06  (-0.43,0.44) | 0.05  (-0.45,0.43) |
| C:N | -0.04  (-0.32,0.28) | -0.15  (-0.51,0.24) | 0.58 (0.32,1.3) | 0.24  (-0.24,0.63) | 0.04  (-0.39,0.49) | 0.26  (-0.28,0.57) |
| C:P | -0.02  (-0.29,0.25) | -0.02  (-0.44,0.26) | 0.1  (-0.21,0.51) | 0.48 (0.24,1.03) | -0.07  (-0.46,0.42) | 0.19  (-0.35,0.58) |
| Boldness | -0.08  (-0.42,0.14) | -0.05  (-0.33,0.36) | 0.01  (-0.34,0.35) | -0.03  (-0.31,0.33) | 0.55 (0.26,1.06) | 0.16  (-0.28,0.58) |
| Feeding rate | -0.02  (-0.28,0.3) | 0.03  (-0.39,0.33) | 0.02  (-0.18,0.52) | 0.1  (-0.27,0.43) | 0.05  (-0.25,0.44) | 0.48  (0.29,1.1) |
| b) Within-mesocosms matrix | | | | | | |
|  | AGR | Excretion rate | C:N | C:P | Boldness | Feeding rate |
| AGR | 0.34 (0.25,0.49) | **0.28 (0.01,0.45)** | -0.03  (-0.23,0.24) | 0.12  (-0.07,0.37) | -0.05  (-0.23,0.23) | -0.08  (-0.3,0.15) |
| Excretion rate | 0.09  (-0.01,0.21) | 0.49 (0.33,0.69) | -0.05  (-0.35,0.13) | 0  (-0.27,0.23) | -0.19  (-0.4,0.04) | -0.1  (-0.3,0.18) |
| C:N | -0.01  (-0.12,0.14) | -0.03  (-0.24,0.08) | 0.73 (0.51,1.01) | -0.07  (-0.29,0.17) | **-0.24**  **(-0.46,-0.03)** | -0.09  (-0.29,0.17) |
| C:P | 0.09  (-0.05,0.24) | -0.03  (-0.2,0.18) | -0.06  (-0.25,0.16) | 0.96 (0.71,1.34) | -0.13  (-0.31,0.15) | 0.1  (-0.13,0.33) |
| Boldness | 0.02  (-0.14,0.12) | -0.09  (-0.28,0.03) | -0.2  (-0.39,-0.02) | -0.11  (-0.29,0.14) | 0.75 (0.52,1.03) | 0.18  (-0.07,0.37) |
| Feeding rate | -0.04  (-0.19,0.1) | -0.04  (-0.24,0.12) | -0.05  (-0.26,0.15) | 0.08  (-0.15,0.34) | 0.16  (-0.05,0.36) | 0.95 (0.65,1.3) |

| Table S.B.3. **a) Variance matrix for among** mesocosms and b) variance-covariance matrix within mesocosms (i.e., among-individual) obtained from the multivariate mixed effects Model 4. Diagonal shows trait variances, with between-trait covariances below (pale grey) and the corresponding correlation coefficients above. Estimates and 95 % CI (in brackets) are obtained from posterior distributions of the multivariate mixed effects model. Significant 95% CI are highlighted in bold, while marginally significant CI are in italics (overlap with zero by < **0.05**). | | | | | | |
| --- | --- | --- | --- | --- | --- | --- |
| a) Among-mesocosms matrix | | | | | | |
|  | AGR | Excretion rate | C:N | C:P | Boldness | Feeding rate |
| AGR | 0.28  (0.21, 0.64) | NA | NA | NA | NA | NA |
| Excretion rate | NA | 0.6  (0.3, 1.02) | NA | NA | NA | NA |
| C:N | NA | NA | 0.48  (0.27, 0.98) | NA | NA | NA |
| C:P | NA | NA | NA | 0.36  (0.23, 0.77) | NA | NA |
| Boldness | NA | NA | NA | NA | 0.41  (0.23, 0.81) | NA |
| Feeding rate | NA | NA | NA | NA | NA | 0.36  (0.25, 0.85) |
| b) Within-mesocosms matrix | | | | | | |
|  | AGR | Excretion rate | C:N | C:P | Boldness | Feeding rate |
| AGR | 0.36  (0.25, 0.49) | **0.24**  **(0.02, 0.48)** | 0.05  (-0.23, 0.24) | 0.11  (-0.08, 0.37*)* | 0.01  (-0.23, 0.22) | -0.12  (-0.34, 0.12) |
| Excretion rate | 0.09  (-0.01, 0.21) | 0.46  (0.34, 0.68) | -0.17  (-0.35, 0.14) | 0  (-0.25, 0.24) | *-0.17*  *(-0.43, 0.03)* | -0.05  (-0.31, 0.16) |
| C:N | -0.03  (-0.13, 0.13) | -0.08  (-0.22, 0.1) | 0.71  (0.52, 1.03) | -0.04  (-0.28, 0.19) | **-0.27**  **(-0.47, -0.03)** | -0.05  (-0.29, 0.16) |
| C:P | 0.07  (-0.05, 0.25) | -0.04  (-0.19, 0.18) | -0.08  (-0.28, 0.16) | 0.96  (0.68, 1.35) | -0.1  (-0.32, 0.11) | 0.11  (-0.12, 0.32) |
| Boldness | 0  (-0.13, 0.13) | -0.08  (-0.28, 0.02) | -0.19  (-0.37, 0) | -0.1  (-0.28, 0.12) | 0.69  (0.51, 1.03) | *0.2*  *(-0.05, 0.4)* |
| Feeding rate | -0.02  (-0.21, 0.08) | -0.03  (-0.23, 0.11) | -0.09  (-0.26, 0.15) | 0.1  (-0.11, 0.35) | 0.11  (-0.03, 0.38) | 0.96  (0.66, 1.3) |

**Supplementary information C: Among-trait covariation**

*Complete pooled data set*

All traits were highly variable between individuals (Fig. S.C.1). Absolute growth rate (AGR) varied from -5.2 to 35.8 mg week^-1^ (mean 8.3 ± 8.4 SD). Boldness ranged from 0 to 255 seconds (52.3 ± 59.8), while feeding rate varied from 0 to 4.6 bites min^-1^ (1.2 ± 1.1). Ammonium excretion rate was ranging from 10.9 to 70.2 μg g^-3/4^ h^-1^ (27.5 ± 13.3). C:N and C:P body ratios ranged from 4.1 to 5.5 (4.4 ± 0.3) and from 27.5 to 74.1 (42.2 ± 8.4), respectively.

Figure S.C.1. Histogram of distribution of each trait: Absolute Growth Rate, ammonium excretion rate, C:N and C:P body ratios, boldness (square root-transformed) and feeding rate (square root-transformed) measured for each fish in the pooled data set (n = 86), as well as, Pearson coefficients and associated significance of correlations between those traits. Red square-*P* < 0.08; ^*^*P* < 0.05; ^**^*P* < 0.01; ^***^*P* < 0.001. For variance-covariance matrices see table 1 in main text).

1. *Within-population comparison in different contexts*

Differences within populations were evaluated for the different contexts (e.g., *D*_fast in high vs low light_ = *I*_fast&high light_ – *I*_fast&low light_ under differing light intensity, competitor intensity etc). Fig 2 (main text) is a simple visual representation of the main difference in each context, while Table S.C.1 contains all the results.

| Table S.C.1. Within-population difference (*D*) variance-covariance matrices comparing the (a-c) fast life history population and the (d-f) slow life history population between contexts (competitor presence vs. absence, high vs. low light intensity, males vs. females). Diagonal shows differences in trait variances (i.e., traces, in grey) and between-trait covariances are below. Estimates are pairwise covariance and 95% CI (in brackets) are estimated from parametric bootstrapping (5000 simulations). Significant 95% CI are highlighted in bold. | | | | | | |
| --- | --- | --- | --- | --- | --- | --- |
| **a) Fast LH in competitor presence vs. absence** | | | | | | |
|  | AGR | Excretion rate | C:N | C:P | Boldness | Feeding rate |
| AGR | -0.37 (-0.74, 0.08) | NA | NA | NA | NA | NA |
| Excretion rate | -0.08 (-0.53, 0.4) | -0.73 (-1.44, 0.15) | NA | NA | NA | NA |
| C:N | 0.1 (-0.34, 0.52) | 0.46 (-0.14, 1.01) | 0.85 (-0.53, 2.26) | NA | NA | NA |
| C:P | -0.18 (-0.56, 0.22) | 0.51 (-0.12, 1.09) | -0.48 (-0.94, 0.03) | 0.81 (-0.24, 1.97) | NA | NA |
| Boldness | -0.33 (-0.82, 0.12) | **0.96 (0.33, 1.47)** | -0.13 (-0.64, 0.37) | 0.13 (-0.36, 0.64) | -0.14 (-0.88, 0.68) | NA |
| Feeding rate | -0.17 (-0.69, 0.34) | 0.49 (-0.25, 1.14) | 0.31 (-0.17, 0.82) | 0.03 (-0.48, 0.55) | -0.19 (-0.94, 0.53) | -0.21 (-0.87, 0.44) |
| **b) Fast LH in high vs. low light** | | | | | | |
|  | AGR | Excretion rate | C:N | C:P | Boldness | Feeding rate |
| AGR | -0.12 (-0.68, 0.45) | NA | NA | NA | NA | NA |
| Excretion rate | 0.46 (-0.07, 0.9) | -0.23 (-1.01, 0.66) | NA | NA | NA | NA |
| C:N | 0.06 (-0.48, 0.57) | -0.18 (-0.92, 0.49) | 0.17 (-1.43, 1.99) | NA | NA | NA |
| C:P | 0.21 (-0.22, 0.56) | 0.2 (-0.35, 0.76) | 0.25 (-0.23, 0.64) | 0.74 (-0.42, 2.1) | NA | NA |
| Boldness | 0.24 (-0.3, 0.76) | 0.06 (-0.61, 0.73) | -0.36 (-0.89, 0.17) | -0.06 (-0.61, 0.54) | **-0.93 (-1.64,-0.13)** | NA |
| Feeding rate | 0.19 (-0.35, 0.78) | 0.12 (-0.54, 0.82) | 0.34 (-0.2, 0.87) | -0.22 (-0.74, 0.29) | -0.36 (-1.16, 0.42) | -0.19 (-0.84, 0.43) |
| **c) Fast LH in males vs. females** | | | | | | |
|  | AGR | Excretion rate | C:N | C:P | Boldness | Feeding rate |
| AGR | **-0.64 (-1.08,-0.19)** | NA | NA | NA | NA | NA |
| Excretion rate | 0.07 (-0.33, 0.46) | 0.18 (-0.7, 1) | NA | NA | NA | NA |
| C:N | -0.17 (-0.58, 0.29) | -0.53 (-1.12, 0.07) | 1.19 (-0.37, 2.82) | NA | NA | NA |
| C:P | -0.07 (-0.48, 0.38) | -0.06 (-0.69, 0.6) | 0.35 (-0.14, 0.86) | -0.25 (-1.38, 0.81) | NA | NA |
| Boldness | **0.53 (0.05, 0.97)** | -0.18 (-0.86, 0.47) | -0.35 (-0.95, 0.2) | -0.54 (-1.07, 0.03) | -0.25 (-0.93, 0.58) | NA |
| Feeding rate | 0.12 (-0.32, 0.52) | 0.66 (-0.02, 1.32) | 0.03 (-0.58, 0.58) | 0 (-0.53, 0.54) | -0.46 (-1.14, 0.32) | 0.19 (-0.55, 0.92) |
| **d) Slow LH in competitor presence vs. absence** | | | | | | |
|  | AGR | Excretion rate | C:N | C:P | Boldness | Feeding rate |
| AGR | -0.3 (-1.41, 0.66) | NA | NA | NA | NA | NA |
| Excretion rate | -0.41 (-1.03, 0.23) | -0.58 (-1.53, 0.37) | NA | NA | NA | NA |
| C:N | -0.2 (-1.04, 0.8) | -0.14 (-0.48, 0.24) | 0.63 (-0.2, 1.46) | NA | NA | NA |
| C:P | 0.28 (-0.56, 1.19) | -0.27 (-0.73, 0.18) | 0.3 (-0.47, 1.17) | 0.19 (-0.68, 1.1) | NA | NA |
| Boldness | **0.62 (0.1, 1.15)** | 0.06 (-0.32, 0.49) | 0.23 (-0.15, 0.6) | 0.08 (-0.31, 0.47) | -0.37 (-0.76, 0.11) | NA |
| Feeding rate | 0.5 (-0.15, 1.08) | -0.28 (-0.81, 0.3) | 0.25 (-0.16, 0.64) | -0.05 (-0.47, 0.4) | 0.03 (-0.36, 0.4) | -0.39 (-0.95, 0.21) |
| **e) Slow LH in high vs. low light** | | | | | | |
|  | AGR | Excretion rate | C:N | C:P | Boldness | Feeding rate |
| AGR | 0.01 (-1.07, 1.04) | NA | NA | NA | NA | NA |
| Excretion rate | -0.54 (-1.19, 0.08) | -1.18 (-2.34, 0.02) | NA | NA | NA | NA |
| C:N | 0.03 (-0.82, 0.9) | -0.28 (-0.64, 0.07) | 0.52 (-0.38, 1.34) | NA | NA | NA |
| C:P | 0.09 (-0.72, 0.98) | -0.49 (-1, 0.02) | 0.6 (-0.12, 1.36) | -0.19 (-0.97, 0.69) | NA | NA |
| Boldness | 0.39 (-0.25, 0.94) | 0.19 (-0.25, 0.66) | 0.15 (-0.25, 0.53) | 0.34 (-0.16, 0.8) | -0.48 (-0.93, 0.04) | NA |
| Feeding rate | 0.05 (-0.65, 0.74) | 0.14 (-0.45, 0.7) | 0.21 (-0.21, 0.65) | -0.09 (-0.6, 0.37) | 0.12 (-0.36, 0.56) | -0.21 (-0.79, 0.38) |
| **f) Slow LH in males vs. females** | | | | | | |
|  | AGR | Excretion rate | C:N | C:P | Boldness | Feeding rate |
| AGR | **-0.9 (-1.71, -0.08)** | NA | NA | NA | NA | NA |
| Excretion rate | **-0.62 (-1.11,-0.11)** | -0.12 (-1.16, 1) | NA | NA | NA | NA |
| C:N | **-0.7 (-1.27, -0.03)** | **-0.24 (-0.47,-0.02)** | -0.23 (-1.08, 0.66) | NA | NA | NA |
| C:P | -0.55 (-1.14, 0.11) | **-0.6 (-1.08, -0.09)** | -0.41 (-1.09, 0.23) | -0.79 (-1.56, 0) | NA | NA |
| Boldness | **0.67 (0.18, 1.11)** | 0.23 (-0.15, 0.6) | 0 (-0.39, 0.36) | 0.26 (-0.22, 0.75) | -0.17 (-0.64, 0.34) | NA |
| Feeding rate | 0.23 (-0.4, 0.81) | **-0.61 (-1.11,-0.02)** | -0.1 (-0.44, 0.24) | 0.15 (-0.33, 0.6) | -0.18 (-0.53, 0.19) | -0.35 (-0.92, 0.23) |

1. *Between-population comparison*

Table S.C.2. Difference (*D*) variance-covariance matrices for comparisons of fast vs. slow life histories and b) males vs. females. Diagonal shows differences in trait variances (i.e., traces, in grey) and between-trait covariances are below. Brackets contain 95% confidence intervals. Estimates are pairwise covariance and confidence intervals are estimated from parametric bootstrapping (5000 simulations). Bold mark 95% confidence intervals that do not overlap with zero, while italics mark those than only overlap with zero with less than **0.05**.

|  | | | | | | |
| --- | --- | --- | --- | --- | --- | --- |
|  | **a) Fast vs. slow life history population** | | | | | |
|  | AGR | Excretion rate | C:N | C:P | Boldness | Feeding rate |
| AGR | **-0.65 (-1.26, -0.02)** |  |  |  |  |  |
| Excretion rate | 0.01 (-0.46, 0.43) | 0.22 (-0.52, 0.86) |  |  |  |  |
| C:N | **-0.65 (-1.19, -0.14)** | **-0.38 (-0.74, -0.03)** | 0.39 (-0.52, 1.33) |  |  |  |
| C:P | *-0.4 (-0.88, 0.04)* | -0.07 (-0.49, 0.32) | -0.28 (-0.74, 0.19) | 0.01 (-0.71, 0.78) |  |  |
| Boldness | 0.2 (-0.24, 0.62) | -0.01 (-0.41, 0.43) | 0.24 (-0.1, 0.56) | 0.14 (-0.21, 0.52) | **0.55 (0.08, 1.02)** |  |
| Feeding rate | -0.02 (-0.46, 0.46) | 0.13 (-0.29, 0.59) | 0 (-0.34, 0.32) | -0.07 (-0.41, 0.27) | 0.31 (-0.16, 0.76) | 0.35 (-0.08, 0.8*)* |
| **b) Males vs. females** | | | | | | |
|  | AGR | Excretion rate | C:N | C:P | Boldness | Feeding rate |
| AGR | **-0.74 (-1.22, -0.28)** |  |  |  |  |  |
| Excretion rate | -0.26 (-0.6, 0.06) | 0.03 (-0.67, 0.65) |  |  |  |  |
| C:N | *-0.35 (-0.74, 0.04)* | **-0.36 (-0.7, -0.02)** | 0.63 (-0.37, 1.8) |  |  |  |
| C:P | -0.29 (-0.68, 0.11) | -0.34 (-0.76, 0.05) | -0.05 (-0.48, 0.34) | -0.55 (-1.32, 0.13) |  |  |
| Boldness | **0.52 (0.2, 0.84)** | 0.03 (-0.38, 0.43) | -0.25 (-0.6, 0.1) | -0.08 (-0.41, 0.34) | -0.22 (-0.72, 0.33) |  |
| Feeding rate | 0.16 (-0.22, 0.52) | 0 (-0.44, 0.43) | -0.13 (-0.47, 0.24) | 0.04 (-0.3, 0.38) | -0.23 (-0.65, 0.2) | -0.07  (-0.53, 0.4) |

Figure S.C.2. Difference in trait variance-covariance (estimates ± 95% CI) comparing a) the fast life history population with the slow life history population, and b) males with females. Significant differences are in black. AGR = absolute growth rate, Bold = Boldness, CN = C:N body ratio, CP = C:P body ratio, Exc = Ammonium excretion rate, and Feed = Feeding rate.

**Supplementary information D: Mean and variance values of variation with in the functional POLSs**

The cosine squared (cos^2^) indicates how well an element (individual or variable) is represented in a PC axis and is evaluated using the squared cosine between the vector issued from the element considered and its projection on the axis. Specifically, cos^2^ closed to 1 indicates that the element is well projected on the axis.

Significant differences between a treatment level and the overall mean along the PC axes were tested using *v*-test, where absolute *v*-test values larger than 1.96 indicate significant differences (Lê *et al.*, 2008).

| Table S.D.1. PCA results for supplementary categorical treatments for the first two principal components (PC 1 and PC 2) for fish traits. *cos*^2^ refers to the quality of the projection of the category on a PC axis (value close to 1 indicates good projection), *v*-test is a test value that indicates whether treatment levels are significantly different (\|*v*-test\| >1.96) or not (\|*v*-test\| < 1.96), and η^2^ is the square correlation ratio and analogous to *R*^2^ from multiple linear regression. Significant *P* values are highlighted in bold. | | | | | | | | |
| --- | --- | --- | --- | --- | --- | --- | --- | --- |
|  | PC 1 | | | | PC 2 | | | |
| Categories | Coordinate estimate | *cos*^2^ | *v*-test | *P* | Coordinate estimate | *cos*^2^ | *v*-test | *P* |
| Slow LH | -0.33 | 0.71 | -2.26 | **0.023** | -0.05 | 0.02 | -0.38 | 0.708 |
| Fast LH | 0.33 | 0.71 | 2.26 | **0.023** | 0.05 | 0.02 | 0.38 | 0.708 |
| Low light | 0.30 | 0.58 | 2.07 | **0.038** | 0.19 | 0.24 | 1.56 | 0.120 |
| High light | -0.30 | 0.58 | -2.07 | **0.038** | -0.19 | 0.24 | -1.56 | 0.120 |
| No competitor | 0.35 | 0.41 | 2.39 | **0.016** | -0.35 | 0.41 | -2.77 | **0.005** |
| Competitor | -0.34 | 0.41 | -2.39 | **0.016** | 0.34 | 0.41 | 2.77 | **0.005** |
| Female | 0.60 | 0.81 | 4.27 | **< 0.001** | 0.01 | 0.00 | 0.05 | 0.957 |
| Male | -0.63 | 0.81 | -4.27 | **< 0.001** | -0.01 | 0.00 | -0.05 | 0.957 |
|  | η^2^ | | *P* | | η^2^ | | *P* | |
| Sex | 0.21 | | **< 0.001** | | 0.00 | | 0.96 | |
| Competitor | 0.07 | | **0.02** | | 0.09 | | **0.005** | |
| Life history | 0.06 | | **0.02** | | 0.00 | | 0.71 | |
| Light exposure | 0.05 | | **0.04** | | 0.03 | | 0.12 | |

Figure S.D.1. Pearson correlation tests (coefficients and significance; ^*^*P* < 0.05; ^**^*P* < 0.01; ^***^*P* < 0.001) performed between mean (between-mesocosm variation) and variance (noted Var; within-mesocosm variation) values of fish PC scores (PC 1 and PC 2) in each mesocosm (dots; n = 24). The frequency distribution of each measure is also displayed using histogram.

**Supplementary information E: Effects of functional POLS vs. experimental treatment on invertebrate abundances and ecosystem processes**

Overall, intraspecific trait variation (both between- and within-mesocosm) affected invertebrate abundances (Table S.E.1, Fig. S.E.1) but not ecosystem processes (Table S.E.2), the latter being mainly affected by light intensity (Table S.E.4).

| Table S.E.1. Effect of fish functional POLS (**variance on PC 1 and, average and variance on PC 2)** on invertebrate abundances given by model estimates, standard error, *z* and *P* values. Significant *P* adjusted for multiple comparisons (FDR-adj-*P*) are highlighted in bold. Each invertebrate abundance was tested independently using a mixed effects model with negative binomial distribution. Fish PC scores were standardised to zero mean and standard deviation units to ease interpretation. Estimates refer to abundances in number of individuals. | | | | | |
| --- | --- | --- | --- | --- | --- |
|  | Estimate | Std. Error | *z* | *P* | FDR-adj-*P* |
| **Cyclopidae** |  |  |  |  |  |
| (Intercept) | 5.8 | 0.31 | 5.68 | < 0.001 | < 0.001 |
| Variance PC 1 | 0.8 | 0.46 | -0.62 | 0.539 | 0.819 |
| Average PC 2 | 0.4 | 0.34 | -2.72 | 0.007 | **0.025** |
| Variance PC 2 | 1.3 | 0.31 | 0.88 | 0.381 | 0.740 |
| **Calanidae** |  |  |  |  |  |
| (Intercept) | 6.6 | 0.59 | 3.21 | 0.001 | 0.006 |
| Variance PC 1 | 0.6 | 0.86 | -0.56 | 0.576 | 0.819 |
| Average PC 2 | 2.0 | 0.89 | 0.76 | 0.449 | 0.763 |
| Variance PC 2 | 0.7 | 0.70 | -0.42 | 0.672 | 0.834 |
| **Bosminidae** |  |  |  |  |  |
| (Intercept) | 4.4 | 0.31 | 4.77 | < 0.001 | < 0.001 |
| Variance PC 1 | 0.5 | 0.44 | -1.66 | 0.096 | 0.276 |
| Average PC 2 | 0.4 | 0.30 | -2.92 | 0.003 | **0.014** |
| Variance PC 2 | 1.3 | 0.34 | 0.88 | 0.379 | 0.740 |
| **Chironomidae** |  |  |  |  |  |
| (Intercept) | 69.3 | 0.43 | 9.97 | < 0.001 | < 0.001 |
| Variance PC 1 | 0.7 | 0.47 | -0.76 | 0.448 | 0.763 |
| Average PC 2 | 0.7 | 0.43 | -0.89 | 0.373 | 0.740 |
| Variance PC 2 | 0.8 | 0.59 | -0.38 | 0.703 | 0.834 |
| **Planorbidae** |  |  |  |  |  |
| (Intercept) | 3.2 | 0.16 | 7.00 | < 0.001 | < 0.001 |
| Variance PC 1 | 0.8 | 0.19 | -1.23 | 0.219 | 0.514 |
| Average PC 2 | 0.5 | 0.16 | -4.79 | < 0.001 | **< 0.001** |
| Variance PC 2 | 0.6 | 0.18 | -3.10 | 0.002 | **0.008** |
| **Corbiculidae** |  |  |  |  |  |
| (Intercept) | 8.2 | 0.12 | 17.61 | < 0.001 | < 0.001 |
| Variance PC 1 | 1.3 | 0.12 | 2.11 | 0.035 | 0.119 |
| Average PC 2 | 0.9 | 0.12 | -0.67 | 0.500 | 0.810 |
| Variance PC 2 | 0.9 | 0.13 | -0.81 | 0.417 | 0.763 |
| **Ostracoda** |  |  |  |  |  |
| (Intercept) | 2.9 | 0.18 | 6.04 | < 0.001 | < 0.001 |
| Variance PC 1 | 1.3 | 0.16 | 1.66 | 0.097 | 0.276 |
| Average PC 2 | 1.0 | 0.18 | 0.11 | 0.912 | 0.944 |
| Variance PC 2 | 1.0 | 0.19 | -0.08 | 0.936 | 0.944 |
| **Nematoda** |  |  |  |  |  |
| (Intercept) | 83.8 | 0.23 | 19.02 | < 0.001 | < 0.001 |
| Variance PC 1 | 0.9 | 0.22 | -0.56 | 0.578 | 0.819 |
| Average PC 2 | 1.1 | 0.24 | 0.41 | 0.680 | 0.834 |
| Variance PC 2 | 1.5 | 0.25 | 1.64 | 0.101 | 0.276 |
| **Hydrachnidia** |  |  |  |  |  |
| (Intercept) | 20.8 | 0.29 | 10.60 | < 0.001 | < 0.001 |
| Variance PC 1 | 0.9 | 0.30 | -0.38 | 0.702 | 0.834 |
| Average PC 2 | 1.1 | 0.26 | 0.44 | 0.660 | 0.834 |
| Variance PC 2 | 2.7 | 0.37 | 2.64 | 0.008 | **0.030** |

Figure S.E.1. Change in invertebrate abundances with within-mesocosms variance in medaka’s second functional POLS (Variance PC 2). PC 2 is positively correlated with fish C:N body ratio and feeding rate and negatively correlated with ammonium excretion rate (Table 1). Solid lines represent significant predicted changes.

| Table S.E.2. Effect of fish functional POLS (**variance on PC 1 and, average and variance on PC 2**) on ecosystem processes given by model estimates, standard error, *t* and *P* values. Significant *P* adjusted for multiple comparisons (FDR-adj-*P*) are highlighted in bold. Each ecosystem process was tested independently using a generalized linear mixed effects model. Pelagic algae stock was log-transformed. Fish PC scores were standardised to zero mean and standard deviation units to ease interpretation. | | | | | | |
| --- | --- | --- | --- | --- | --- | --- |
|  | Estimate | | Std. Error | *t* | *P* | FDR-adj-*P* |
| **Sediment C:N ratio** |  | |  |  |  |  |
| (Intercept) | 16.16 | | 0.13 | 125.94 | 0.000 | 0.000 |
| Variance PC 1 | -0.04 | | 0.13 | -0.27 | 0.788 | 0.893 |
| Average PC 2 | 0.08 | | 0.13 | 0.64 | 0.532 | 0.819 |
| Variance PC 2 | -0.05 | | 0.13 | -0.36 | 0.723 | 0.834 |
| **Sediment C:P ratio** |  | |  |  |  |  |
| (Intercept) | 1233.38 | | 17.47 | 70.61 | 0.000 | 0.000 |
| Variance PC 1 | -7.92 | | 18.11 | -0.44 | 0.667 | 0.834 |
| Average PC 2 | -15.12 | | 17.94 | -0.84 | 0.409 | 0.763 |
| Variance PC 2 | 3.54 | | 18.16 | 0.19 | 0.847 | 0.929 |
| **SRP concentration** (μg L^-1^) |  | |  |  |  |  |
| (Intercept) | 9.65 | | 0.53 | 18.07 | 0.053 | 0.171 |
| Variance PC 1 | 0.16 | | 0.38 | 0.42 | 0.682 | 0.834 |
| Average PC 2 | 0.06 | | 0.37 | 0.16 | 0.874 | 0.929 |
| Variance PC 2 | -0.34 | | 0.37 | -0.92 | 0.368 | 0.740 |
| **N concentration** (μg L^-1^) |  | |  |  |  |  |
| (Intercept) | 16.34 | | 2.18 | 7.49 | 0.086 | 0.267 |
| Variance PC 1 | 0.23 | | 0.44 | 0.52 | 0.607 | 0.834 |
| Average PC 2 | -0.61 | | 0.42 | -1.44 | 0.166 | 0.419 |
| Variance PC 2 | 0.49 | | 0.42 | 1.18 | 0.254 | 0.576 |
| **Pelagic algae stock** (logged µg L^-1^) | |  |  |  |  |  |
| (Intercept) | 1.19 | | 0.09 | 12.90 | < 0.001 | < 0.001 |
| Variance PC 1 | -0.02 | | 0.10 | -0.20 | 0.841 | 0.929 |
| Average PC 2 | 0.03 | | 0.09 | 0.36 | 0.722 | 0.834 |
| Variance PC 2 | 0.10 | | 0.10 | 1.09 | 0.289 | 0.633 |
| **Respiration** (CR24; mg O_2_) |  | |  |  |  |  |
| (Intercept) | 110.21 | | 13.79 | 7.99 | < 0.001 | < 0.001 |
| Variance PC 1 | -2.42 | | 14.31 | -0.17 | 0.867 | 0.929 |
| Average PC 2 | -5.46 | | 14.17 | -0.39 | 0.704 | 0.834 |
| Variance PC 2 | -8.31 | | 14.34 | -0.58 | 0.569 | 0.819 |
| **GPP (**mg O_2_) |  | |  |  |  |  |
| (Intercept) | 153.63 | | 16.67 | 9.22 | 0.113 | 0.295 |
| Variance PC 1 | -1.50 | | 15.30 | -0.10 | 0.923 | 0.944 |
| Average PC 2 | -11.76 | | 15.04 | -0.78 | 0.443 | 0.763 |
| Variance PC 2 | -10.84 | | 15.15 | -0.72 | 0.483 | 0.801 |
| **Benthic algae stock** (µg cm^-2^) |  | |  |  |  |  |
| (Intercept) | 0.16 | | 0.02 | 10.60 | < 0.001 | < 0.001 |
| Variance PC 1 | 0.02 | | 0.02 | 1.36 | 0.190 | 0.461 |
| Average PC 2 | 0.00 | | 0.02 | -0.07 | 0.944 | 0.944 |
| Variance PC 2 | -0.01 | | 0.02 | -0.58 | 0.569 | 0.819 |

| Table S.E.3. Effect of mesocosm manipulation (populations with different life histories, light intensity and competitor) on invertebrate abundances given by model estimates, standard error, *z* and *P* values. Significant *P* adjusted for multiple comparisons (FDR-adj-*P*) are highlighted in bold. Each invertebrate abundance was tested independently using a mixed effects model with negative binomial distribution. Intercept refers to slow life history, low light density and absence of competitor. Estimates refer to abundances in number of individuals. | | | | | |
| --- | --- | --- | --- | --- | --- |
|  | *Estimate* | *Std. Error* | *z* | *P* | *FDR-adj-P* |
| **Cyclopidae** |  |  |  |  |  |
| (Intercept) | 12.0 | 0.75 | 3.32 | 0.001 | 0.005 |
| Fast life history | 3.5 | 0.84 | 1.48 | 0.140 | 0.250 |
| High light intensity | 0.3 | 0.92 | -1.15 | 0.250 | 0.379 |
| Competitor presence | 0.3 | 0.76 | -1.72 | 0.085 | 0.206 |
| **Calanidae** |  |  |  |  |  |
| (Intercept) | 5.5 | 1.65 | 1.04 | 0.298 | 0.431 |
| Fast life history | 2.1 | 1.56 | 0.48 | 0.631 | 0.715 |
| High light intensity | 1.0 | 1.18 | 0.01 | 0.996 | 0.996 |
| Competitor presence | 0.7 | 1.56 | -0.25 | 0.804 | 0.868 |
| **Bosminidae** |  |  |  |  |  |
| (Intercept) | 3.3 | 0.81 | 1.46 | 0.145 | 0.253 |
| Fast life history | 1.7 | 0.78 | 0.70 | 0.482 | 0.591 |
| High light intensity | 2.3 | 0.73 | 1.14 | 0.254 | 0.379 |
| Competitor presence | 0.9 | 0.77 | -0.16 | 0.871 | 0.925 |
| **Chironomidae** | |  |  |  |  |
| (Intercept) | 1.6 | 0.43 | 1.14 | 0.256 | 0.379 |
| Fast life history | 1.0 | 0.43 | -0.06 | 0.956 | 0.996 |
| High light intensity | 135.9 | 0.46 | 10.58 | < 0.001 | **< 0.001** |
| Competitor presence | 0.5 | 0.42 | -1.59 | 0.111 | 0.225 |
| **Planorbidae** | |  |  |  |  |
| (Intercept) | 5.2 | 0.54 | 3.05 | 0.002 | 0.010 |
| Fast life history | 0.7 | 0.50 | -0.81 | 0.417 | 0.545 |
| High light intensity | 1.4 | 0.47 | 0.69 | 0.487 | 0.591 |
| Competitor presence | 0.8 | 0.50 | -0.41 | 0.683 | 0.761 |
| **Corbiculidae** | |  |  |  |  |
| (Intercept) | 7.0 | 0.28 | 6.99 | < 0.001 | < 0.001 |
| Fast life history | 1.3 | 0.24 | 0.95 | 0.344 | 0.468 |
| High light intensity | 1.4 | 0.25 | 1.28 | 0.202 | 0.319 |
| Competitor presence | 0.8 | 0.24 | -0.96 | 0.338 | 0.468 |
| **Ostracoda** |  |  |  |  |  |
| (Intercept) | 3.6 | 0.32 | 3.92 | < 0.001 | 0.001 |
| Fast life history | 0.6 | 0.36 | -1.51 | 0.132 | 0.242 |
| High light intensity | 1.8 | 0.37 | 1.57 | 0.115 | 0.225 |
| Competitor presence | 0.6 | 0.37 | -1.43 | 0.153 | 0.260 |
| **Nematoda** |  |  |  |  |  |
| (Intercept) | 26.1 | 0.39 | 8.29 | < 0.001 | < 0.001 |
| Fast life history | 1.0 | 0.42 | 0.04 | 0.972 | 0.996 |
| High light intensity | 3.7 | 0.41 | 3.18 | 0.001 | **0.008** |
| Competitor presence | 2.0 | 0.42 | 1.59 | 0.113 | 0.225 |
| **Hydrachnidia** | |  |  |  |  |
| (Intercept) | 13.5 | 0.52 | 4.98 | < 0.001 | < 0.001 |
| Fast life history | 3.0 | 0.57 | 1.91 | 0.056 | 0.152 |
| High light intensity | 0.2 | 0.60 | -2.35 | 0.019 | 0.070 |
| Competitor presence | 3.2 | 0.60 | 1.91 | 0.056 | 0.152 |

| Table S.E.4. Effect of mesocosm manipulation (life history population, light intensity and competitor) on ecosystem processes given by model estimates, standard error, *t,* degrees of freedom, d.f., and *P* values. Significant *P* adjusted for multiple comparisons (FDR-adj-*P*) are highlighted in bold. Each ecosystem process was tested independently using a generalized linear mixed effects model. Pelagic algae stock was log-transformed. Intercept refers to slow life history, low light density and absence of competitor. | | | | | | | | |
| --- | --- | --- | --- | --- | --- | --- | --- | --- |
|  | Estimate | | Std. Error | | d.f. | *t* | *P* | *FDR-adj-P* |
| **Sediment C:N ratio** | | |  | |  |  |  |  |
| (Intercept) | 15.43 | | 0.17 | | 20.00 | 89.58 | < 0.001 | < 0.001 |
| Fast life history | 0.29 | | 0.17 | | 20.00 | 1.69 | 0.106 | 0.225 |
| High light intensity | 0.60 | | 0.17 | | 20.00 | 3.49 | 0.002 | **0.010** |
| Competitor presence | 0.57 | | 0.17 | | 20.00 | 3.31 | 0.003 | **0.014** |
| **Sediment C:P ratio** | | |  | |  |  |  |  |
| (Intercept) | 1273.09 | | 31.36 | | 20.00 | 40.59 | < 0.001 | < 0.001 |
| Fast life history | 22.25 | | 31.36 | | 20.00 | 0.71 | 0.486 | 0.591 |
| High light intensity | -60.19 | | 31.36 | | 20.00 | -1.92 | 0.069 | 0.175 |
| Competitor presence | -41.49 | | 31.36 | | 20.00 | -1.32 | 0.201 | 0.319 |
| **SRP concentration** (μg L^-1^) | |  |  | |  |  |  |  |
| (Intercept) | 9.57 | | 0.80 | | 5.28 | 11.90 | < 0.001 | < 0.001 |
| Fast life history | 0.26 | | 0.71 | | 19.00 | 0.36 | 0.720 | 0.789 |
| High light intensity | 0.44 | | 0.71 | | 19.00 | 0.62 | 0.545 | 0.640 |
| Competitor presence | -0.54 | | 0.71 | | 19.00 | -0.76 | 0.458 | 0.588 |
| **N concentration** (μg L^-1^) | | |  | |  |  |  |  |
| (Intercept) | 16.40 | | 2.01 | | 1.21 | 8.16 | 0.053 | 0.152 |
| Fast life history | 0.00 | | 0.70 | | 19.00 | 0.01 | 0.995 | 0.996 |
| High light intensity | 1.65 | | 0.70 | | 19.00 | 2.37 | 0.029 | 0.093 |
| Competitor presence | -1.78 | | 0.70 | | 19.00 | -2.55 | 0.020 | 0.071 |
| **Pelagic algae stock** (logged µg L^-1^) | | | |  |  |  |  |  |
| (Intercept) | 1.20 | | 0.17 | | 20.00 | 6.88 | < 0.001 | < 0.001 |
| Fast life history | 0.11 | | 0.17 | | 20.00 | 0.61 | 0.546 | 0.640 |
| High light intensity | -0.28 | | 0.17 | | 20.00 | -1.62 | 0.120 | 0.226 |
| Competitor presence | 0.16 | | 0.17 | | 20.00 | 0.91 | 0.376 | 0.501 |
| **Respiration** (CR24 mg O_2_) | | |  | |  |  |  |  |
| (Intercept) | 35.72 | | 20.02 | | 7.29 | 1.78 | 0.116 | 0.225 |
| Fast life history | 31.70 | | 18.81 | | 19.00 | 1.69 | 0.108 | 0.225 |
| High light intensity | 73.82 | | 18.81 | | 19.00 | 3.92 | 0.001 | **0.005** |
| Competitor presence | 43.47 | | 18.81 | | 19.00 | 2.31 | 0.032 | 0.100 |
| **GPP** (mg O_2_) |  | |  | |  |  |  |  |
| (Intercept) | 82.18 | | 25.00 | | 4.94 | 3.29 | 0.022 | 0.075 |
| Fast life history | 29.87 | | 21.78 | | 19.00 | 1.37 | 0.186 | 0.309 |
| High light intensity | 76.27 | | 21.78 | | 19.00 | 3.50 | 0.002 | **0.010** |
| Competitor presence | 36.77 | | 21.78 | | 19.00 | 1.69 | 0.108 | 0.225 |
| **Benthic algae stock** (µg cm^-2^) | | |  | |  |  |  |  |
| (Intercept) | 0.31 | | 0.08 | | 20.00 | 4.09 | 0.001 | 0.004 |
| Fast life history | -0.06 | | 0.03 | | 20.00 | -1.94 | 0.067 | 0.175 |
| High light intensity | -0.01 | | 0.03 | | 20.00 | -0.51 | 0.617 | 0.711 |
| Competitor presence | -0.03 | | 0.03 | | 20.00 | -1.00 | 0.331 | 0.468 |

**Supplementary information F: Effect of fish absence vs. presence**

| Table S.F.1. Effect of absence of fish relative to presence (slow or fast life history fish) on invertebrate abundances and ecosystem processes given by model estimates, standard error, *t,* degrees of freedom, d.f., and *P* values. Significant *P* adjusted for multiple comparisons (FDR-adj-*P*) are highlighted in bold. Each ecosystem process was tested independently using a generalized linear mixed effects model. Pelagic algae stock was log-transformed. Intercept (Int) refers to slow life history, low light density and absence of competitor. | | | | | | | | |
| --- | --- | --- | --- | --- | --- | --- | --- | --- |
|  | Estimate | | Std. Err | | z | *P* | FDR-adj-*P* | |
| **Cyclopidae (number individuals)** | | | | | | | | |
| Int: Presence slow LH | 5.25 | | 0.41 | | 4.07 | 0.000 | 0.000 | |
| Presence fast LH fish | 2.43 | | 0.57 | | 1.56 | 0.118 | 0.208 | |
| Fish absence | 6.84 | | 0.56 | | 3.41 | 0.001 | **0.002** | |
| **Calanidae (number individuals)** | | | | | | | | |
| Int: Presence slow LH | 4.08 | | 0.90 | | 1.57 | 0.117 | 0.208 | |
| Presence fast LH fish | 2.71 | | 1.26 | | 0.79 | 0.429 | 0.534 | |
| Fish absence | 0.10 | | 1.34 | | -1.71 | 0.088 | 0.172 | |
| **Bosmidae (number individuals)** | | | | | | | | |
| Int: Presence slow LH | 4.25 | | 0.51 | | 2.83 | 0.005 | 0.010 | |
| Presence fast LH fish | 2.43 | | 0.71 | | 1.24 | 0.213 | 0.316 | |
| Fish absence | 1.47 | | 0.72 | | 0.54 | 0.591 | 0.701 | |
| **Chironomidae (number individuals)** | | | | | | | | |
| Int: Presence slow LH | 56.08 | | 0.04 | | 104.46 | 0.000 | 0.000 | |
| Presence fast LH fish | 1.89 | | 0.05 | | 13.35 | 0.000 | **0.000** | |
| Fish absence | 1.23 | | 0.05 | | 4.04 | 0.000 | **0.000** | |
| **Planorbidae (number individuals)** | | | | | | | | |
| Int: Presence slow LH | 5.58 | | 0.31 | | 5.60 | 0.000 | 0.000 | |
| Presence fast LH fish | 0.69 | | 0.44 | | -0.85 | 0.395 | 0.504 | |
| Fish absence | 0.49 | | 0.45 | | -1.57 | 0.117 | 0.208 | |
| **Corbiculidae (number individuals)** | | | | | | | | |
| Int: Presence slow LH | 7.50 | | 0.18 | | 11.02 | 0.000 | 0.000 | |
| Presence fast LH fish | 1.26 | | 0.25 | | 0.90 | 0.371 | 0.485 | |
| Fish absence | 1.26 | | 0.25 | | 0.90 | 0.371 | 0.485 | |
| **Ostracoda (number individuals)** | | | | | | | | |
| Int: Presence slow LH | 3.75 | | 0.26 | | 5.03 | 0.000 | 0.000 | |
| Presence fast LH fish | 0.62 | | 0.39 | | -1.22 | 0.223 | 0.316 | |
| Fish absence | 0.58 | | 0.39 | | -1.40 | 0.163 | 0.268 | |
| **Nematoda (number individuals)** | | | | | | | | |
| Int: Presence slow LH | 83.00 | | 0.31 | | 14.07 | 0.000 | 0.000 | |
| Presence fast LH fish | 1.24 | | 0.44 | | 0.48 | 0.628 | 0.713 | |
| Fish absence | 0.19 | | 0.45 | | -3.74 | 0.000 | **0.000** | |
| **Hydrachnidia (number individuals)** | | | | | | | | |
| Int: Presence slow LH | 18.00 | | 0.47 | | 6.20 | 0.000 | 0.000 | |
| Presence fast LH fish | 2.24 | | 0.66 | | 1.22 | 0.221 | 0.316 | |
| Fish absence | 0.88 | | 0.66 | | -0.19 | 0.846 | 0.880 | |
| **Sediment C:N ratio** | | | | | | | | |
| Int: Presence slow LH | 16.02 | 0.19 | | 83.79 | | 0.000 | | 0.000 |
| Presence fast LH fish | 0.29 | 0.27 | | 1.08 | | 0.288 | | 0.397 |
| Fish absence | 0.53 | 0.27 | | 1.95 | | 0.059 | | 0.121 |
| **Sediment C:P ratio** | | | | | | | | |
| Int: Presence slow LH | 1222.25 | 32.25 | | 37.90 | | 0.000 | | 0.000 |
| Presence fast LH fish | 22.25 | 45.61 | | 0.49 | | 0.629 | | 0.713 |
| Fish absence | -5.72 | 45.61 | | -0.13 | | 0.901 | | 0.919 |
| **SRP concentration (µg L^-1^)** | | | | | | | | |
| Int: Presence slow LH | 9.52 | 0.49 | | 19.33 | | 0.000 | | 0.000 |
| Presence fast LH fish | 0.26 | 0.70 | | 0.37 | | 0.712 | | 0.790 |
| Fish absence | -0.19 | 0.70 | | -0.28 | | 0.784 | | 0.833 |
| **N concentration (µg L^-1^)** | | | | | | | | |
| Int: Presence slow LH | 16.34 | 1.35 | | 12.11 | | 0.000 | | 0.000 |
| Presence fast LH fish | 0.00 | 1.91 | | 0.00 | | 0.998 | | 0.998 |
| Fish absence | -4.61 | 1.91 | | -2.41 | | 0.021 | | **0.046** |
| **Pelagic algae stock (logged µg L^-1^)** | | | | | | | | |
| Int: Presence slow LH | 1.14 | 0.10 | | 10.94 | | 0.000 | | 0.000 |
| Presence fast LH fish | 0.11 | 0.15 | | 0.73 | | 0.471 | | 0.572 |
| Fish absence | -0.74 | 0.15 | | -5.01 | | 0.000 | | **0.000** |
| **Respiration (CR, mg O_2_)** | | | | | | | | |
| Int: Presence slow LH | 110.48 | 17.44 | | 6.34 | | 0.000 | | 0.000 |
| Presence fast LH fish | 37.15 | 24.66 | | 1.51 | | 0.141 | | 0.241 |
| Fish absence | -110.40 | 24.66 | | -4.48 | | 0.000 | | **0.000** |
| **GPP (mg O_2_)** | | | | | | | | |
| Int: Presence slow LH | 193.94 | 24.79 | | 7.82 | | 0.000 | | 0.000 |
| Presence fast LH fish | 48.44 | 35.06 | | 1.38 | | 0.176 | | 0.281 |
| Fish absence | -193.69 | 35.06 | | -5.52 | | 0.000 | | **0.000** |
| **Benthic algae stock (µg cm^-2^)** | | | | | | | | |
| Int: Presence slow LH | 0.19 | 0.03 | | 6.27 | | 0.000 | | 0.000 |
| Presence fast LH fish | -0.05 | 0.04 | | -1.30 | | 0.204 | | 0.316 |
| Fish absence | -0.01 | 0.04 | | -0.29 | | 0.771 | | 0.833 |

**References**

Bott TL (2006) Primary productivity and community respiration. In: Methods in Stream Ecology, 2nd Edition. Elsevier, New York, pp 263–290.

Burnham KP, Anderson DR (2002) Model Selection and Inference: A Practical Information-Theoretical Approach, 2nd Edition. Springer-Verlag, New York.

Dingemanse NJ, Dochtermann NA (2013) Quantifying individual variation in behaviour: mixed-effect modelling approaches. *Journal of Animal Ecology* 82: 39–54.

Hadfield JD (2010) MCMC Methods for Multi-Response Generalized Linear Mixed Models: The MCMCglmm *R* Package. *J Stat Soft* 33. doi:10.18637/jss.v033.i02

Harmon LJ, Matthews B, Des Roches S, Chase JM, Shurin JB, Schluter D (2009) Evolutionary diversification in stickleback affects ecosystem functioning. *Nature* 458: 1167–1170.

Houslay TM, Vierbuchen M, Grimmer AJ, Young AJ, Wilson AJ (2018) Testing the stability of behavioural coping style across stress contexts in the Trinidadian guppy. *Functional Ecology* 32: 424–438.

Houslay TM, Wilson AJ (2017) Avoiding the misuse of BLUP in behavioural ecology. *Behav Ecol* 28: 948–952.

Paris-Palacios S, Biagianti-Risbourg S (2006) Hepatocyte nuclear structure and subcellular distribution of copper in zebrafish \emphBrachydanio rerio and roach \emphRutilus rutilus (Teleostei, Cyprinidae) exposed to copper sulphate. *Aquatic Toxicology* 77: 306–313.

Spiegelhalter DJ, Best NG, Carlin BP, van der Linde A (2002) Bayesian measures of model complexity and fit. *J Royal Statistical Soc B* 64: 583–639.

Torres LE, Vanni MJ (2007) Stoichiometry of nutrient excretion by fish: interspecific variation in a hypereutrophic lake. *Oikos* 116: 259–270.

Vanni MJ, Flecker AS, Hood JM, Headworth JL (2002) Stoichiometry of nutrient recycling by vertebrates in a tropical stream: linking species identity and ecosystem processes. *Ecology Letters* 5: 285–293.

Whiles MR, Huryn AD, Taylor BW, Reeve JD (2009) Influence of handling stress and fasting on estimates of ammonium excretion by tadpoles and fish: recommendations for designing excretion experiments. *Limnology and Oceanography-Methods* 7: 1–7.
